# Identification of NOTCH4 mutation as a response biomarker for immune checkpoint inhibitor therapy

**DOI:** 10.1186/s12916-021-02031-3

**Published:** 2021-07-21

**Authors:** Junyu Long, Dongxu Wang, Xu Yang, Anqiang Wang, Yu Lin, Mingjun Zheng, Haohai Zhang, Xinting Sang, Hanping Wang, Ke Hu, Haitao Zhao

**Affiliations:** 1 Department of Liver Surgery, State Key Laboratory of Complex Severe and Rare Diseases, Peking Union Medical College Hospital, Chinese Academy of Medical Sciences & Peking Union Medical College, Beijing, China; 2 Department of Gastrointestinal Surgery, Key Laboratory of Carcinogenesis and Translational Research, Ministry of Education, Peking University Cancer Hospital & Institute, Beijing, China; 3 Shenzhen Withsum Technology Limited, Shenzhen, China; 4 Department of Obstetrics and Gynecology, University Hospital, LMU Munich, Maistrasse 11, 80337 Munich, Germany; 5 Liver Center and The Transplant Institute, Department of Medicine, Beth Israel Deaconess Medical Center, Harvard Medical School, Boston, MA USA; 6 Division of Pulmonary and Critical Care Medicine, State Key Laboratory of Complex Severe and Rare Diseases, Peking Union Medical College Hospital, Chinese Academy of Medical Sciences & Peking Union Medical College, Beijing, China; 7 Department of Radiation Oncology, Peking Union Medical College Hospital, Peking Union Medical College & Chinese Academy of Medical Sciences, Beijing, China

**Keywords:** NOTCH4, Immune checkpoint inhibitor, Clinical benefit, Objective response rate

## Abstract

**Background:**

Immune checkpoint inhibitor (ICI) therapy elicits durable antitumor responses in patients with many types of cancer. Genomic mutations may be used to predict the clinical benefits of ICI therapy. NOTCH homolog-4 (NOTCH4) is frequently mutated in several cancer types, but its role in immunotherapy is still unclear. Our study is the first to study the association between NOTCH4 mutation and the response to ICI therapy.

**Methods:**

We tested the predictive value of NOTCH4 mutation in the discovery cohort, which included non-small cell lung cancer, melanoma, head and neck squamous cell carcinoma, esophagogastric cancer, and bladder cancer patients, and validated it in the validation cohort, which included non-small cell lung cancer, melanoma, renal cell carcinoma, colorectal cancer, esophagogastric cancer, glioma, bladder cancer, head and neck cancer, cancer of unknown primary, and breast cancer patients. Then, the relationships between NOTCH4 mutation and intrinsic and extrinsic immune response mechanisms were studied with multiomics data.

**Results:**

We collected an ICI-treated cohort (n = 662) and found that patients with NOTCH4 mutation had better clinical benefits in terms of objective response rate (ORR: 42.9% vs 25.9%, P = 0.007), durable clinical benefit (DCB: 54.0% vs 38.1%, P = 0.021), progression-free survival (PFS, hazard ratio [HR] = 0.558, P < 0.001), and overall survival (OS, HR = 0.568, P = 0.006). In addition, we validated the prognostic value of NOTCH4 mutation in an independent ICI-treated cohort (n = 1423). Based on multiomics data, we found that NOTCH4 mutation is significantly associated with enhanced immunogenicity, including a high tumor mutational burden, the expression of costimulatory molecules, and activation of the antigen-processing machinery, and NOTCH4 mutation positively correlates activated antitumor immunity, including infiltration of diverse immune cells and various immune marker sets.

**Conclusions:**

Our findings indicated that NOTCH4 mutation serves as a novel biomarker correlated with a better response to ICI therapy.

**Supplementary Information:**

The online version contains supplementary material available at 10.1186/s12916-021-02031-3.

## Background

Immune checkpoint inhibitors (ICIs) targeting the programmed cell death (ligand) 1 [PD-(L)1] and cytotoxic T lymphocyte antigen 4 (CTLA-4) pathways have emerged as treatment strategies for various types of cancers [1, 2]. Nevertheless, only a few patients have achieved a lasting response to ICI therapy in clinical practice. As a consequence, biomarkers predicting response may assist in identifying patients who will benefit the most from ICI therapy. It has been reported that some emerging biomarkers can serve to predict the therapeutic effect, such as the expression of programmed cell death ligand-1 (PD-L1) on cancer cells and antigen-presenting cells, which can direct the inflammatory tumor microenvironment (TME) and the tumor mutational burden (TMB), leading to an increase in the expression of tumor-specific neoantigens. Nevertheless, current biomarkers still have many shortcomings; for example, many biomarkers exhibit intra/intertumor heterogeneity, the cost of treatment is too high, the predictive ability is not satisfactory, and the cutoff value is not standardized, limiting the application of these biomarkers in clinical practice. Therefore, there is an urgent need to discover more predictive biomarkers.

Notch signaling, as an evolutionarily conserved pathway, plays a critical role in tissue homeostasis, fetal development, and organogenesis [3]. Notch signaling is aberrantly activated in different cancer types, such as lung, prostate, cervical, colon, pancreatic, and breast cancer, renal carcinoma, and large-cell and Hodgkin lymphomas [4]. Notch signaling can be a tumor-suppressive or an oncogenic mechanism depending on the tissue microenvironment [4]. In certain cases, the Notch receptor has a paradoxical effect or reaction on the progression or clinicopathological characteristics of a specific cancer. To date, four Notch receptors (Notch 1, Notch 2, Notch 3, and Notch 4) have been identified in mammals [5]. Notch signaling critically affects the tumorigenesis and proliferation control of cells in the gastrointestinal tract [6]. It is interesting to note that Notch signaling acts as an oncogenic event in some cancers and a cancer suppressor in others. Growing evidence shows that Notch signaling is related to antitumor immunity/immunotherapy [7, 8]. For example, in murine models of autoimmune diseases, pharmacological inhibition of NOTCH1 signaling can reduce the number of activated T helper type 1 cells [7, 8]. Furthermore, preclinical evidence has revealed that the tumor microenvironment and cancer cells evolve various mechanisms to evade T cell-mediated killing, including the suppression of NOTCH signaling. Consistent with the importance of NOTCH in regulating the immune response against cancers, chimeric antigen receptor T cells generated with synthetic NOTCH receptors exhibit specific and potent cytotoxic responses [9, 10]. Moreover, NOTCH mutation is thought to be enriched in non-small cell lung cancer (NSCLC) in smokers [11]. Mazzotta et al. found that co-occurring mutations in NOTCH1-3 and homologous repair genes were associated with a durable clinical benefit [12]. To date, there is no detailed evidence in clinical practice regarding whether NOTCH4 mutation affects the response to ICI therapy.

In the current analysis, we systematically collected and integrated a large amount of clinical and genomic data to evaluate the predictive value of NOTCH4 mutation. We found that NOTCH4 mutation was predictive of improved progression-free survival (PFS), longer overall survival (OS), a better objective response rate (ORR), and a higher durable clinical benefit (DCB) of ICI therapy.

## Methods

### The integration of clinical cohorts

**Fig. 1:**
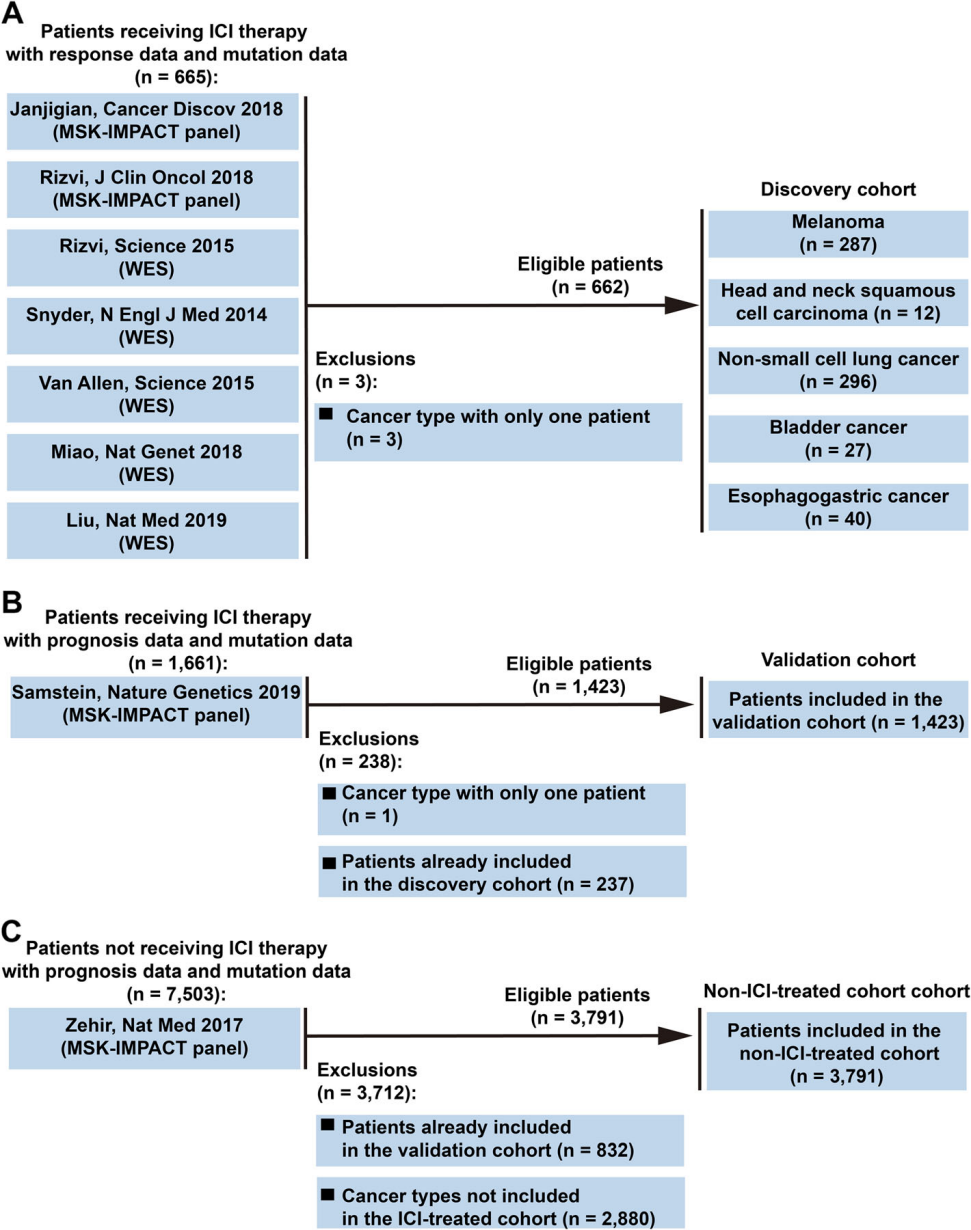
Flowchart of the process used for screening of the study population. **A** Flowchart of the process used for screening of patients included in the discovery cohort. **B** Flowchart of the process used for screening of patients included in the validation cohort. **C** Flowchart of the process used for screening of patients included in the non-ICI-treated cohort

To evaluate the predictive value of NOTCH4 mutation, we collected and integrated a discovery cohort with mutational data and response data from patients treated with ICI therapy based on seven published studies (Fig. 1A) [13–19]. The processed mutational data were obtained from cBioPortal (https://www.cbioportal.org). All nonsynonymous mutations, including translation start site, splice site, nonstop, nonsense, frameshift, and missense mutations, were considered for inclusion in the study [20]. Tumors with and without nonsynonymous somatic mutations of NOTCH4 were defined as NOTCH4-mutant (NOTCH4-MUT) and NOTCH4-wildtype (NOTCH4-WT), respectively. Memorial Sloan Kettering-Integrated Mutation Profiling of Actionable Cancer Targets (MSK-IMPACT) panel, an integrated genomic profiling panel authorized by the U.S. Food and Drug Administration (FDA), was employed for sequencing samples from the first two cohorts [13, 14], and whole-exome sequencing (WES) was used for sequencing of samples from the latter five cohorts [15–19]. Cancer types with patient numbers < 10 were excluded. Finally, the discovery cohort included 662 patients with five cancer types: non-small cell lung cancer (NSCLC) (n = 296), melanoma (n = 287), head and neck squamous cell carcinoma (HNSCC) (n = 12), esophagogastric cancer (n = 40), and bladder cancer (n = 27).

Furthermore, we validated the predictive value of NOTCH4 mutation in the validation cohort, an independent ICI-treated cohort from Samstein et al. (n = 1423) that was composed of patients with NSCLC (n=157), melanoma (n = 320), renal cell carcinoma (n=151), colorectal cancer (n=109), esophagogastric cancer (n = 88), glioma (n =117), bladder cancer (n = 215), head and neck cancer (n = 138), cancer of unknown origin (n = 84), and breast cancer (n = 44) and included survival data but no response data (Fig. 1B) [21]. The processed mutational data from Samstein et al. were obtained from cBioPortal (https://www.cbioportal.org).

To determine whether the survival differences between patients with NOTCH4-MUT and NOTCH4-WT tumors are attributable to a general prognostic benefit of NOTCH4-MUT, unrelated to ICI therapy, the non-ICI-treated cohort (n = 3,791) from Zehir et al. was also included (Fig. 1C) [22]. The processed mutational data from Zehir et al. were obtained from cBioPortal (https://www.cbioportal.org).

In addition, data on the mutation profiles (sequenced by WES) and mRNA expression profiles of 10,143 patients with 33 types of cancers in The Cancer Genome Atlas (TCGA) cohort, downloaded from the PanCancer Atlas consortium (https://gdc.cancer.gov/about-data/publications/pancanatlas), were used to investigate the distinct immune response landscapes of NOTCH4-WT and NOTCH4-MUT tumors.

### Calculation of the TMB

For WES-sequenced samples, the TMB was defined as the total number of nonsynonymous mutations divided by the exome size (38 Mb). For MSK-IMPACT panel-sequenced samples, the total number of nonsynonymous mutations was normalized to the exonic coverage of the MSK-IMPACT panel (1.22, 1.06, and 0.98 Mb in the 468-, 410-, and 341-gene panels, respectively).

### Measurement of clinical outcomes

Response Evaluation Criteria in Solid Tumors (RECIST) version 1.1 was employed to assess the ORR. DCB was classified as no durable benefit (NDB, stable disease [SD] that lasted ≤ 6 months or progression of the disease [PD]) or DCB (complete response [CR]/partial response [PR] or SD that lasted > 6 months) [14]. The primary clinical outcomes were ORR, DCB, PFS, and OS. In the non-ICI-treated cohort, OS was calculated from the procedure date when the tumor specimen was collected to the date of death or most recent follow-up [22]. In the ICI-treated cohort, OS was measured from the start date of ICI therapy, and patients who did not die were censored at the date of last contact.

### CIBERSORT immune infiltration estimation

CIBERSORT immune infiltration proportions were acquired from the pancancer immune landscape project conducted by Thorsson et al. [23]. CIBERSORT is a gene expression-based deconvolution algorithm that uses support vector regression to infer cell-type proportions in data from bulk tumor samples of mixed cell types [24]. The proportions of 22 types of infiltrating immune cells (neutrophils, eosinophils, mast cells activated, mast cells resting, dendritic cells activated, dendritic cells resting, macrophages M2, macrophages M1, macrophages M0, monocytes, NK cells activated, NK cells resting, T cells gamma delta, Tregs, T cells follicular helper, T cells CD4 memory activated, T cells CD4 memory resting, T cells CD4 naïve, T cells CD8, plasma cells, B cells memory, and B cells naïve) were calculated via the CIBERSORT algorithm based on normalized gene expression data.

### Leukocyte fraction, lymphocyte fraction, and TIL fraction analyses

The levels of TILs from genomics estimates and the levels of TILs from H&E-stained image estimates in the TCGA pancancer cohort were estimated by analyzing the data from Thorsson et al. and Saltz et al., respectively [23, 25]. The genomics estimate of the TIL fraction was obtained by multiplying an aggregated proportion of the lymphocyte fraction in the immune compartment evaluated by the CIBERSORT method with the leukocyte fraction derived from DNA methylation. The lymphocyte fraction is an aggregation of CIBERSORT estimates of plasma cells, activated and resting NK cells, CD8 T cells, gamma-delta T cells, T regulatory cells, follicular helper T cells, naïve, resting and activated memory CD4 T cells, and naïve and memory B cells. By using deep learning-based lymphocyte classification with convolutional neural networks (CNNs), Saltz et al. presented global mappings of TILs for over 5,000 H&E-stained diagnostic whole-slide images from the TCGA dataset, which represented a benchmark for TIL analysis.

### Microenvironment cell population (MCP) evaluation

The absolute abundance of eight immune (CD3 T cells, CD8 T cells, cytotoxic lymphocytes, NK cells, B lymphocytes, cells originating from monocytes (monocytic lineage), myeloid dendritic cells and neutrophils) and two stromal cell populations (endothelial cells and fibroblasts) was estimated by the MCP-counter method [26]. These results were highly reproducible and concordant with the results obtained in vitro, by using mRNA mixtures, and ex vivo, by using immunohistochemical cell quantifications on paraffin-embedded tissue sections.

### Assessment of immune signatures

Twenty-nine classical immune signatures were obtained from a previous study [27]. The enrichment levels of the 29 immune signatures in each sample were quantified according to the single-sample gene set enrichment analysis (ssGSEA) method using the “GSVA” R package (version: 1.34.0) [28].

### Cytolytic activity score (CYT)

CYT was calculated as the geometric mean of granzyme A (GZMA) and perforin 1 (PRF1) expression [29].

### Calculation of immunogenomic indicators

Immunogenomic indicators were acquired from the pancancer immune landscape project conducted by Thorsson et al. [23]. In brief, neoantigen prediction by SNVs was performed using OptiType tool v1.2, NetMHCpan v3.0, and PanCancer MC3 Consortium [30–32]. Neoantigen prediction by indels was performed using VEP v87 (Ensembl Variant Effect Predictor) and the pVAC-Seq v4.0.8 pipeline with NetMHCpan v3.0 [31, 33]. TCR diversity scores (Shannon entropy and richness) were inferred from tumor RNA-seq data [34, 35].

### Statistical analysis

Fisher’s exact test was used to assess the enrichment of NOTCH4 status with response (ORR and DCB). The log-rank test and Cox proportional hazards regression analysis were used to analyze the difference in PFS and OS between NOTCH4-MUT and NOTCH4-WT patients. Statistical analysis of comparisons between two groups was conducted using the Wilcoxon test. All statistical analyses were conducted with R software (version 3.6.3), and P values were two-tailed. A P value less than 0.05 was considered significant.

## Results

### NOTCH4 mutation was related to better clinical outcomes for ICI therapy

**Fig. 2:**
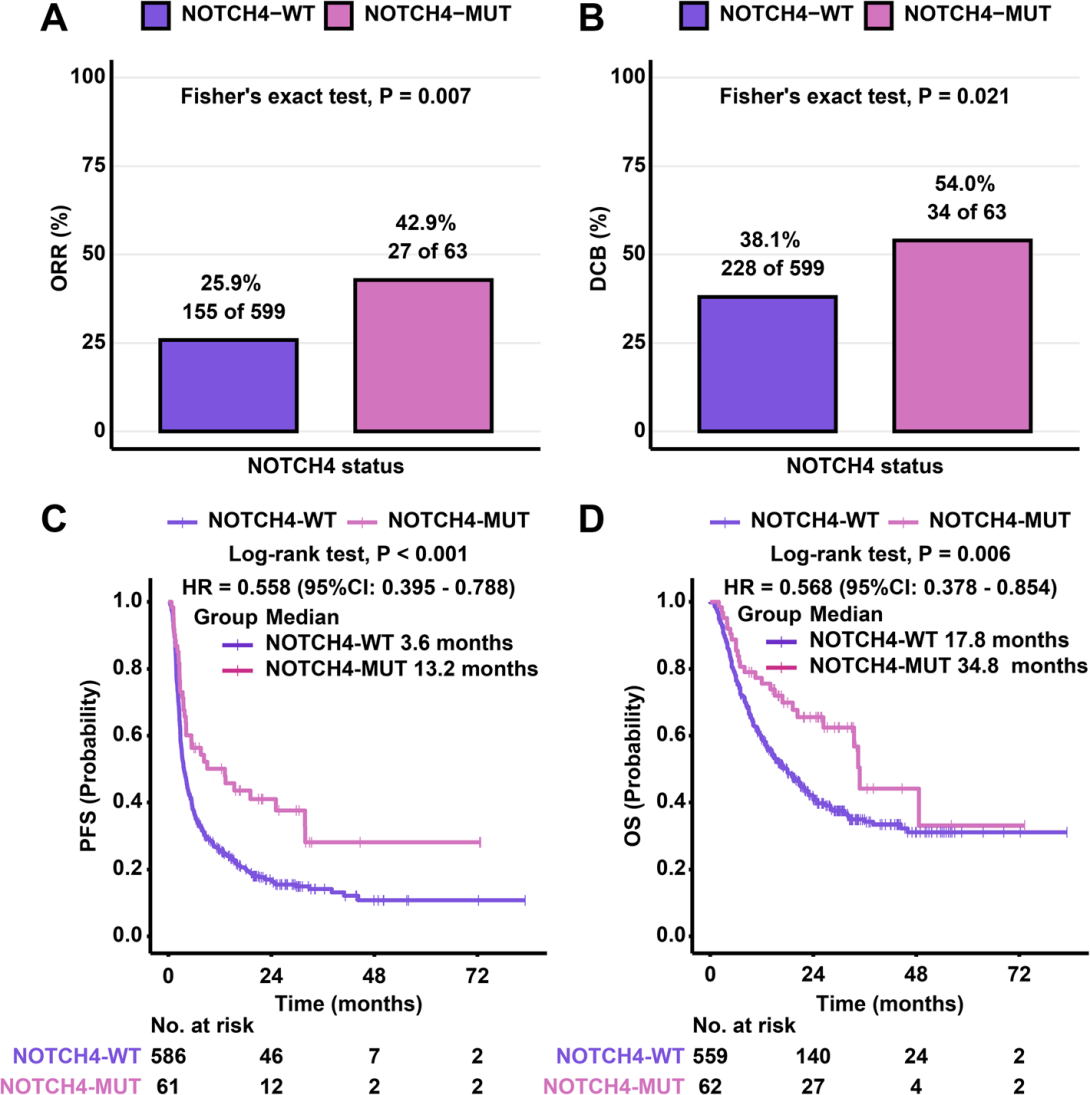
Association of NOTCH4 mutation with clinical outcomes. **A** Histogram showing the proportions of patients who achieved an objective response rate (ORR) in NOTCH4-WT and NOTCH4-MUT tumors. **B** Histogram showing the proportions of patients who achieved a durable clinical benefit (DCB) in NOTCH4-WT and NOTCH4-MUT tumors. **C** Predictive value of NOTCH4 mutation for progression-free survival (PFS) in the discovery cohort. **D** Predictive value of NOTCH4 mutation for overall survival (OS) in the discovery cohort

We integrated the response data and mutational data of 7 ICI-treated cohorts into the discovery cohort, including 662 patients across 5 cancer types: non-small cell lung cancer (NSCLC) (n = 296), melanoma (n = 287), head and neck squamous cell carcinoma (HNSCC) (n = 12), esophagogastric cancer (n = 40), and bladder cancer (n = 27) (Fig. 1A). Table 1 summarizes the clinical characteristics of 662 patients in the discovery cohort. According to RECIST version 1.1, the response data of 662 patients were evaluated in the discovery cohort. The ORR of patients was significantly increased in patients with NOTCH4-MUT (ORR = 42.9%, 27/63) compared to patients with NOTCH4-WT (ORR = 25.9%, 155/599) (P = 0.007, Fig. 2A). In addition, 54.0% (34/63) of patients with NOTCH4-MUT obtained a DCB, in contrast to only 38.1% (228/599) of patients with NOTCH4-WT (P = 0.021, Fig. 2B). As expected, compared to that in the NOTCH4-WT group (n = 61), longer PFS was observed in the NOTCH4-MUT group (n = 586, hazard ratio [HR] = 0.558 [95% CI: 0.395 to 0.788], P < 0.001, Fig. 2C). The median PFS was 13.2 months in the NOTCH4-MUT group versus 3.6 months in the NOTCH4-WT group. In addition, we randomly selected 61 patients from 586 NOTCH4-WT patients and conducted the survival analysis between them and 61 NOTCH4-MUT patients. We conducted 1000 random samplings, and we found that the median PFS (PFS: 3.831 months, 95% CI 2.187–5.475) of 61 NOTCH4-WT patients was shorter than the median PFS (PFS: 13.17 months) of 61 NOTCH4-MUT patients, and the average HR (HR = 0.554, 95% CI 0.419–0.734) was less than 1 (Additional file 1: Table S1). Therefore, we believe that our conclusion may be stable, indicating that NOTCH4 mutation may be a good predictive factor and NOTCH4-MUT patients may benefit more from ICI treatment. The OS benefit was also more prominent in the NOTCH4-MUT group (median OS: 34.8 months) than in the NOTCH4-WT group (median OS: 17.8 months) (HR = 0.568 [95% CI 0.378 to 0.854], P = 0.006, Fig. 2D).

**Table 1:** Summary of the clinical characteristics of the discovery cohort

Characteristics	No. (%)
**No. of patients**	662
**Median age, years (range)**	64 (55–71)
**Gender**	
Female	278 (42)
Male	384 (58)
**Cancer type**	
Bladder cancer	27 (4)
Esophagogastric cancer	40 (6)
Head and neck cancer	12 (2)
Melanoma	287 (43)
Non-small cell lung cancer	296 (45)
**Drug type**	
Monotherapy	608 (92)
Combination therapy	54 (8)
**Treatment best response**	
CR	43 (6)
PD	319 (48)
PR	139 (21)
SD	161 (24)
**Durable clinical benefit**	
Benefit	262 (40)
Nonbenefit	400 (60)
**NOTCH4 status**	
Wildtype	599 (90)
Mutant	63 (10)

Among 296 NSCLC patients, 72 NSCLC patients had complete NOTCH4 mutation status and PD-L1 status and TMB data. The PD-L1 data were obtained from Rizvi et al. [14]. PD-L1 positive was defined as a PD-L1 score (%) is greater than 0. TMB high was defined as TMB greater than the median value. Then, we graphically compared overall survival according to NOTCH4 mutation status, PD-L1 status, and TMB (Additional file 2: Figure S1A-S1C). Compared with NOTCH4-WT patients (median OS: 22.0 months), NOTCH4-MUT patients (median OS: not reached) had a good survival trend (Additional file 2: Figure S1A). In addition, we compared the response of the NOTCH4 MUT/PD-L1-negative/TMB-low patients (n = 2) and the NOTCH4 MUT /PD-L1-positive/TMB-high patients (n = 2) to the ICI treatment, and we found that the two NOTCH4 MUT/PD-L1-negative/TMB-low patients did not respond to ICI treatment (ORR = 0%), while the two NOTCH4 MUT/PD-L1-positive/TMB-high patients responded to ICI treatment (ORR = 100%) (Additional file 2: Figure S1D). The C-index is one of the most commonly used performance measures for survival models [36]. The higher the value of the C-index is, the better the predictive ability of the model [36]. Therefore, we compared the C-index according to NOTCH4 status, PD-L1 status, and TMB, and we found that the predictive power of NOTCH4 status (C-index = 0.571) and PD-L1 status (C-index = 0.581) was almost the same and greater than the predictive power of TMB (C-index = 0.488) (Additional file 2: Figure S1E).

We extracted the histopathological type information of 296 NSCLC patients from the published articles [14, 15, 18], including 233 patients with lung adenocarcinoma, 41 patients with lung squamous cell carcinoma, and 22 patients with other types or patients who were vaguely labeled as having NSCLC. We found that in lung adenocarcinoma, compared with NOTCH4-WT patients (ORR = 21.2%), NOTCH4-MUT patients (ORR = 25.0%) tended to have a better response to ICI treatment (Additional file 3: Figure S2A). At the same time, in lung squamous cell carcinoma, compared with NOTCH4-WT patients (ORR = 17.9%), NOTCH4-MUT patients (ORR = 50.0%) tended to have a better response to ICI treatment (Additional file 3: Figure S2B).

Mazzotta et al. found that co-occurring mutations in NOTCH1-3 and homologous repair genes were associated with increased efficacy of immunotherapy [12]. Therefore, we extracted the homologous repair genes (ATR, ATM, BRCA1, BRCA2, and MUTYH) from Mazzotta et al [12]. The sample in which all genes in the homologous repair pathway were wild type was designated “homologous repair pathway unaltered.” We did a correlation analysis between homologous repair genes and NOTCH4 mutation in the discovery cohort and found that NOTCH4 mutation was also correlated with homologous repair genes (P = 0.001, Fisher’s exact test) (Additional file 3: Figure S2C). Furthermore, according to Mazzotta’s findings and our results, we believe that the whole NOTCH family (including NOTCH1, NOTCH2, NOTCH3, and NOTCH4) mutation may have an effect on immunotherapy. We defined the sample in which all genes in the NOTCH family were wild type as “NOTCH family unaltered” and the sample in which at least one gene in the NOTCH family was mutated as “NOTCH family altered.” We found that patients with the NOTCH family altered (median OS: 33.5 months) had a better OS benefit than patients with the NOTCH family unaltered (median OS: 15.4 months) in the discovery cohort (HR = 0.594 [95% CI: 0.443 to 0.796], P < 0.001, Additional file 3: Figure S2D). In addition, we extracted the expression profile data of patients with lung adenocarcinoma from the TCGA dataset. The genes of the NOTCH pathway are extracted from Sanchez-Vega et al., including 50 activated genes and 21 repressed genes (Additional file 4: Table S2) [37]. The NOTCH pathway score for each sample was defined as the average expression of activated genes minus the average expression of repressed genes. We compared the NOTCH pathway scores between NOTCH4-MUT patients and NOTCH4-WT patients. We found that NOTCH4-MUT patients had higher expression of NOTCH pathway components than did NOTCH4-WT patients (Additional file 3: Figure S2E).

### NOTCH4 mutation status was an independent predictor of immunotherapy prognosis

Univariate and multivariate Cox regression analyses were used to evaluate the independent predictive value of NOTCH4 status. Univariate Cox regression analysis of PFS revealed that NOTCH4 status and cancer type had prognostic value (Additional file 5: Figure S3A). Then, NOTCH4 status and cancer type were subjected to multivariate Cox regression analysis of PFS. Multivariate Cox regression analysis revealed that NOTCH4 status was an independent prognostic factor associated with PFS (Additional file 5: Figure S3A). The same results were seen in the univariate and multivariate Cox regression analyses for OS. After adjusting for cancer type, NOTCH4 status was an independent prognostic factor associated with OS (Additional file 5: Figure S3B).

### Validation of the prognostic value of NOTCH4 status

To further validate the predictive value of NOTCH4 mutation for OS benefit, an independent ICI-treated cohort (n = 1423) was surveyed to determine the relationship between NOTCH4 mutation and OS (Fig. 1B). In this validation cohort, NOTCH4-MUT patients (median OS: 41.0 months) achieved significantly longer OS than NOTCH4-WT patients (median OS: 18.0 months) (HR = 0.693 [95% CI 0.490 to 0.978], P = 0.033, Additional file 6: Figure S4A). To confirm that the OS benefit of ICI therapy in patients with NOTCH4-MUT was not simply attributed to its general prognostic impact, we further assessed the OS difference between NOTCH4-MUT and NOTCH4-WT patients in a non-ICI-treated cohort (Fig. 1C). There was no OS difference between NOTCH4-MUT patients (median OS: 23.6 months) and NOTCH4-WT patients (median OS: 26.3 months) in the non-ICI-treated cohort (HR = 1.084 [95% CI 0.781 to 1.505], P = 0.628, Additional file 6: Figure S4B).

In addition, we did a subgroup analysis both in the discovery cohort and in the validation cohort. For cancer types with greater than 50 samples in the discovery cohort, including NSCLC (n = 296) and melanoma (n = 287) (Additional file 7: Figure S5A-S5B), we found that in these cancer types, compared with NOTCH4-WT patients, NOTCH4-MUT patients had a good prognosis trend. In addition, the HR of NOTCH4 mutation was less than 1, which proves that NOTCH4 mutation may be a protective factor. Furthermore, the median survival time of NOTCH4-MUT patients was longer than that of NOTCH4-WT patients. In NSCLC, HR was < 1 and the median OS of NOTCH4-MUT patients (median OS: not reached) was longer than that of NOTCH4-WT patients (median OS: 18.0 months) (Additional file 7: Figure S5A). In melanoma, HR was < 1 and the median OS of NOTCH4-MUT patients (median OS: 34.8 months) was longer than that of NOTCH4-WT patients (median OS: 22.1 months). For cancer types with greater than 50 samples in the validation cohort, including bladder cancer (n = 215), cancer of unknown origin (n = 84), colorectal cancer (n = 109), esophagogastric cancer (n = 88), glioma (n = 117), head and neck cancer (n = 138), melanoma (n = 320), NSCLC (n = 157), and renal cell carcinoma (n = 151), the result of the validation set was consistent with the result of the training set (Additional file 7: Figure S5C-S5K). For example, in NSCLC, HR was < 1 and the median OS of NOTCH4-MUT patients (median OS: 7.5 months) was longer than that of NOTCH4-WT patients (median OS: 6.0 months) (Additional file 7: Figure S5C). In melanoma, HR was < 1 and the median OS of NOTCH4-MUT patients (median OS: 47.0 months) was longer than that of NOTCH4-WT patients (median OS: 42.0 months) (Additional file 7: Figure S5D).

### Underlying intrinsic immune response mechanisms of NOTCH4-MUT and NOTCH4-WT tumors

**Fig. 3:**
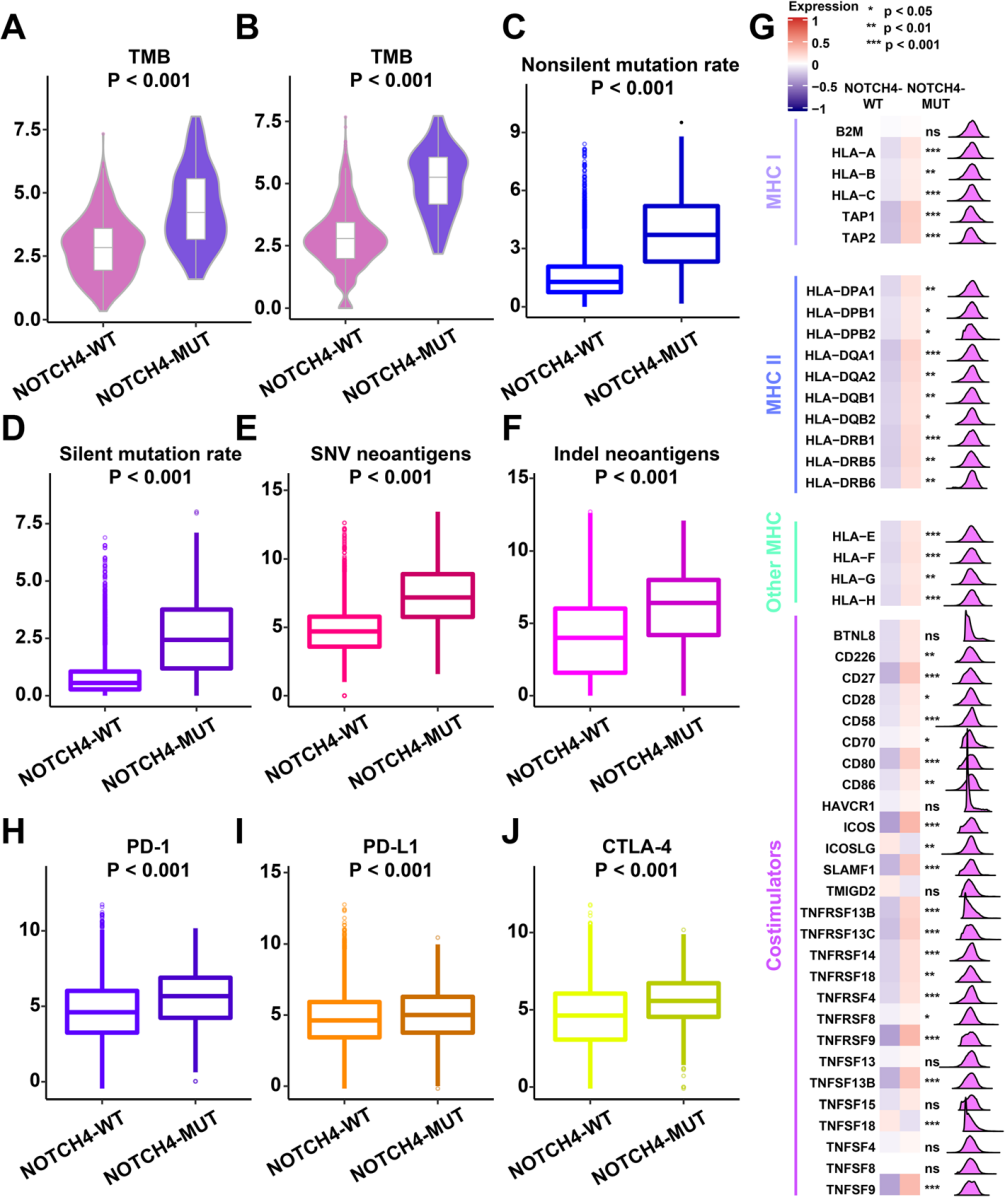
Potential intrinsic immune response landscapes in NOTCH4-WT and NOTCH4-MUT tumors. A Comparison of the TMB between NOTCH4-WT and NOTCH4-MUT tumors in the discovery cohort. **B** Comparison of the TMB between NOTCH4-WT and NOTCH4-MUT tumors in the validation cohort. **C** Comparison of the nonsilent mutation rate between NOTCH4-WT and NOTCH4-MUT tumors in the TCGA cohort. **D** Comparison of the silent mutation rate between NOTCH4-WT and NOTCH4-MUT tumors in the TCGA cohort. **E** Comparison of SNV neoantigens between NOTCH4-WT and NOTCH4-MUT tumors in the TCGA cohort. **F** Comparison of indel neoantigens between NOTCH4-WT and NOTCH4-MUT tumors in the TCGA cohort. **G** Comparison of the expression of MHC molecules and costimulators between NOTCH4-WT and NOTCH4-MUT tumors in the TCGA cohort. **H** Comparison of the expression of PD-1 between NOTCH4-WT and NOTCH4-MUT tumors in the TCGA cohort. **I** Comparison of the expression of PD-L1 between NOTCH4-WT and NOTCH4-MUT tumors in the TCGA cohort. **J** Comparison of the expression of CTLA-4 between NOTCH4-WT and NOTCH4-MUT tumors in the TCGA cohort. Statistical analysis of comparisons between two groups was conducted using the Wilcoxon test

To unravel the potential mechanism underlying the predictive value of NOTCH4 mutation for ICI therapy, the underlying intrinsic immune response mechanisms of NOTCH4-MUT and NOTCH4-WT tumors were investigated. The mechanisms of the innate immune response comprise several major factors: high tumor immunogenicity, the expression of costimulatory molecules, and activation of the antigen-processing machinery (APM). Compared with that in NOTCH4-WT tumors, the TMB was significantly higher in NOTCH4-MUT tumors both in the discovery cohort (P < 0.001, Fig. 3A) and in the validation cohort (P < 0.001, Fig. 3B). In addition, we used multiomics data in the TCGA cohort to explore the differences in immune landscape between NOTCH4-MUT and NOTCH4-WT tumors. Compared to that in NOTCH4-WT tumors, the mutational load (in terms of both the nonsilent mutation rate and the silent mutation rate) (P < 0.001, Fig. 3C, D), and neoantigen load (in terms of both SNV neoantigens and indel neoantigens) (P < 0.001, Fig. 3E, F) were also significantly higher in NOTCH4-MUT tumors, suggesting that NOTCH4-MUT was related to enhanced tumor immunogenicity.

Various processes, including chemotaxis, differentiation, activation, and recognition, are needed for T cell immune function. Disruption of one or several of these processes causes tumor immune escape and T cell dysfunction. First, T cells must successfully identify tumor antigens presented by antigen-presenting cells. The lack of presentation function of major histocompatibility complex (MHC) class I molecules is often one of the main causes of tumor immune escape [38, 39]. Compared to NOTCH4-WT tumors, NOTCH4-MUT tumors had higher expression of MHC I- and II-related antigen-presenting molecules (most P < 0.05, Fig. 3G), which is indicative of stronger immunogenicity.

Another important underlying intrinsic immune response mechanism is the expression of costimulatory molecules. We found that costimulatory molecules were more highly expressed in NOTCH4-MUT tumors than in NOTCH4-WT tumors (most P < 0.05) (Fig. 3G). In addition, compared with NOTCH4-WT tumors, NOTCH4-MUT tumors also showed upregulated expression of immune checkpoint molecules (such as PD-1, PD-L1, and CTLA4) (Fig. 3H–J). These results suggested that NOTCH4-MUT tumors were strongly related to enhanced tumor immunogenicity and a relatively hot immune microenvironment, which firmly supported its predictive value for ICI therapy.

### Underlying extrinsic immune response mechanisms of NOTCH4-MUT and NOTCH4-WT tumors

**Fig. 4:**
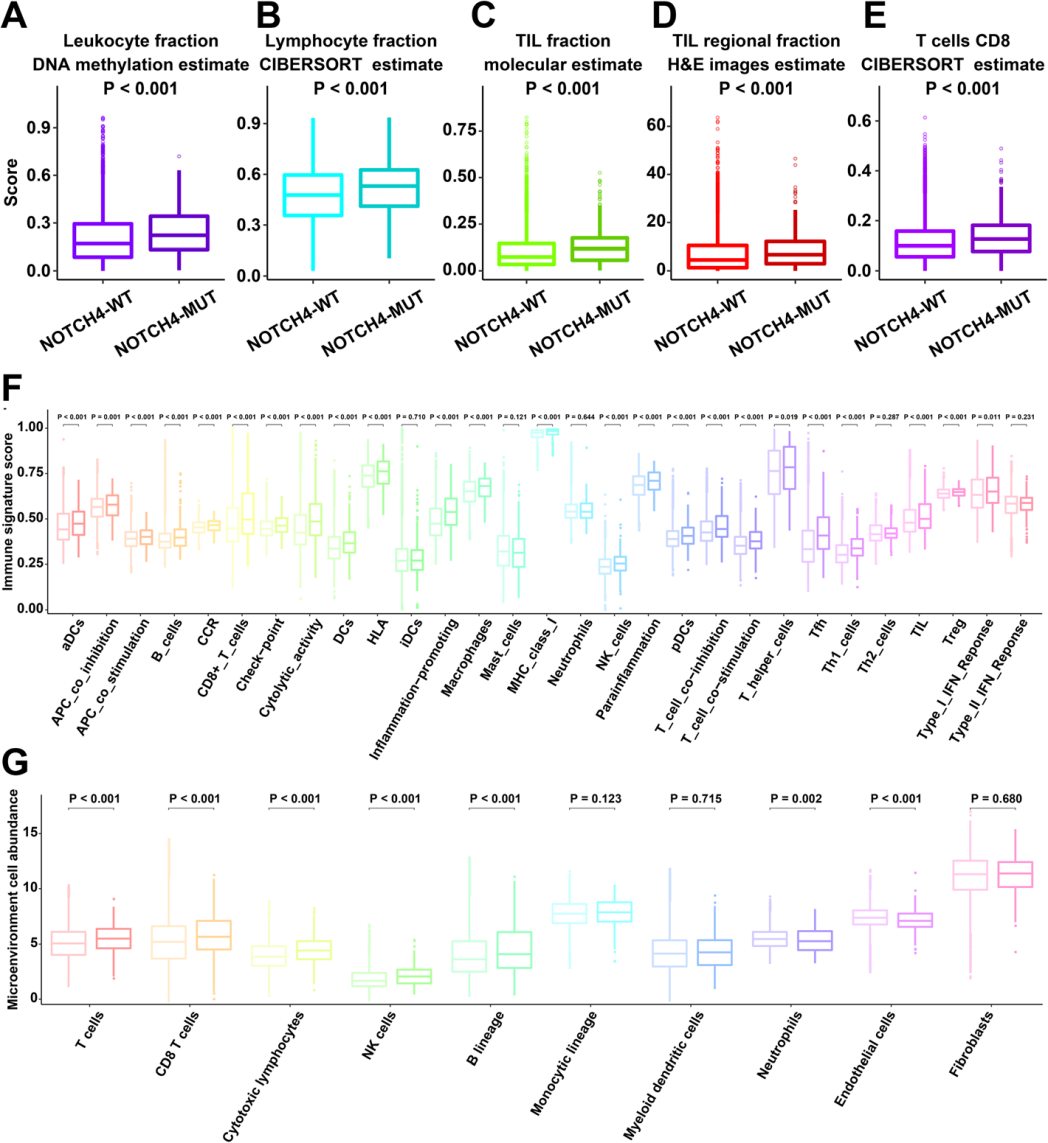
Potential extrinsic immune response landscapes of NOTCH4-WT and NOTCH4-MUT tumors in the TCGA cohort. **A** Comparison of the leukocyte fractions based on DNA methylation data between NOTCH4-WT and NOTCH4-MUT tumors. **B** Comparison of the lymphocyte fractions estimated by the CIBERSORT method based on RNA-sequencing data between NOTCH4-WT and NOTCH4-MUT tumors. **C** Comparison of the TIL fraction based on molecular estimates from processing of cancer genomics data between NOTCH4-WT and NOTCH4-MUT tumors. **D** Comparison of the TIL regional fractions based on estimates from processing diagnostic H&E images between NOTCH4-WT and NOTCH4-MUT tumors. **E** Comparison of CD8 T cells estimated by the CIBERSORT method based on RNA-sequencing data between NOTCH4-WT and NOTCH4-MUT tumors. **F** Comparison of the 29 immune signatures estimated by the ssGSEA method based on RNA-sequencing data between NOTCH4-WT and NOTCH4-MUT tumors. In each immune signature, the light color represents the NOTCH4-WT tumors, and the dark color represents the NOTCH4-MUT tumors. The P value is shown at the top of the graph. **G** Comparison of the 10 cell populations estimated by the MCP-counter method based on RNA-sequencing data between the NOTCH4-WT and NOTCH4-MUT tumors. In each cell type, the light color represents the NOTCH4-WT tumors, and the dark color represents the NOTCH4-MUT tumors. The P value is shown at the top of the graph. Statistical analysis of comparisons between two groups was conducted using the Wilcoxon test

The heterogeneity of TME phenotypes resulted in NOTCH4-MUT and NOTCH4-WT tumors being characterized by distinct immune response landscapes. Several extrinsic components, including the presence of immune cells, high concentrations of immunostimulatory chemokines, higher T cell receptors (TCR) diversity and high cytolytic activity, and the high immunogenicity of cancer cells contribute to the immune response [40, 41]. The infiltration of immune cells into the TME is a prerequisite for antitumor immunity. First, we compared the leukocyte fraction between NOTCH4-WT tumors and NOTCH4-MUT tumors based on DNA methylation arrays and found that NOTCH4-MUT tumors had a larger leukocyte fraction (P < 0.001, Fig. 4A). Second, we compared the fraction of lymphocytes (which is an important cell population in leukocytes) between NOTCH4-WT tumors and NOTCH4-MUT tumors using the CIBERSORT method based on RNA-sequencing data. We obtained similar findings for the high-lymphocyte fraction in NOTCH4-MUT tumors (P < 0.001, Fig. 4B). Third, we compared the molecular estimate of the tumor-infiltrating lymphocyte (TIL) fraction, which was obtained by multiplying an estimate of the leukocyte fraction by an estimate of the lymphocyte fraction within the immune compartment. The comparison showed that the TIL fraction of the NOTCH4-WT tumors was larger than that of the NOTCH4-MUT tumors (P < 0.001, Fig. 4C). Finally, we used the TIL fraction data according to Saltz et al., who used deep learning methods to estimate TILs from hematoxylin and eosin-stained (H&E-stained) slides [25]. Strikingly consistent results were seen for the H&E estimates of the TIL fraction (P < 0.001, Fig. 4D). Therefore, the H&E estimates of the TIL fraction (Fig. 4D) were consistent with molecular estimates of the TIL fraction from molecular genomics assays (Fig. 4C). Specifically, we found that the NOTCH4-MUT tumors had a significantly larger fraction of immune-stimulatory cells (CD8 T cells) (P < 0.001, Fig. 4E). Therefore, from large cell populations to small cell populations and multiomics data, we have proven that the immune cell infiltration of NOTCH4-WT tumors is higher than that of NOTCH4-MUT tumors.

**Fig. 5:**
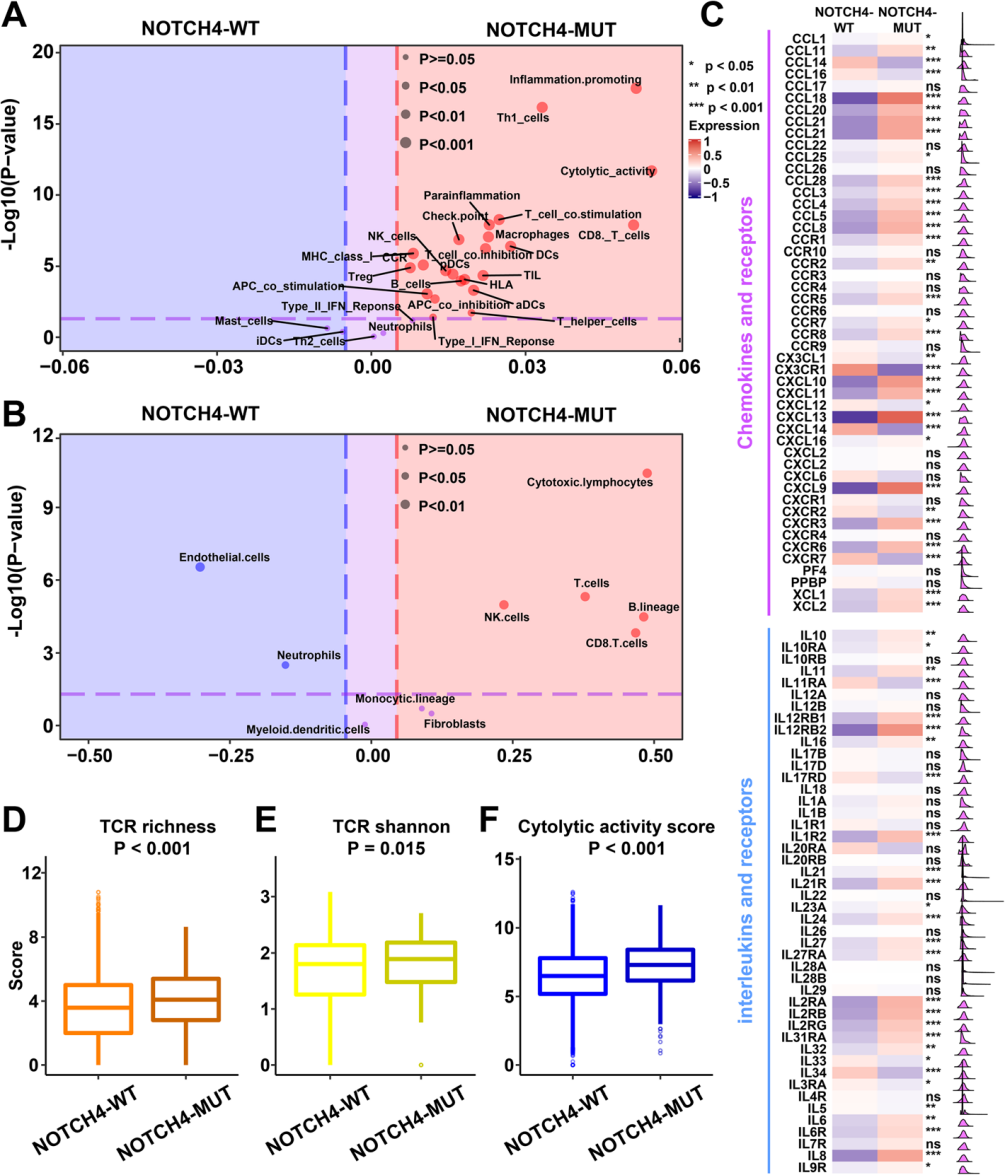
The NOTCH4 mutation was associated with high immune checkpoint expression in the TCGA cohort. **A** Volcano plots of 29 immune signatures estimated by the ssGSEA method based on RNA-sequencing data for NOTCH4-WT tumors and NOTCH4-MUT tumors. Immune signatures enriched in NOTCH4-MUT tumors are marked in red; immune signatures enriched in NOTCH4-WT tumors are marked in blue. **B** Volcano plots of 10 cell populations estimated by the MCP-counter method based on RNA-sequencing data for NOTCH4-WT tumors and NOTCH4-MUT tumors. Cell populations enriched in NOTCH4-MUT tumors are marked in red; cell populations enriched in NOTCH4-WT tumors are marked in blue. **C** Comparison of the expression of chemokines and interleukins between NOTCH4-WT and NOTCH4-MUT tumors. **D** Comparison of the expression of TCR richness between NOTCH4-WT and NOTCH4-MUT tumors. **E** Comparison of the expression of the TCR Shannon index between NOTCH4-WT and NOTCH4-MUT tumors. **F** Comparison of the cytolytic activity score between NOTCH4-WT and NOTCH4-MUT tumors. Statistical analysis of comparisons between two groups was conducted using the Wilcoxon test

To cross-examine the above results with different methods of evaluating immune cells, we analyzed the distribution of immune cells between NOTCH4-WT tumors and NOTCH4-MUT tumors based on other methods of evaluating immune cells. The ssGSEA method was utilized to evaluate the immune status of each patient by analyzing the expression profiles of 29 immune signatures, which represented diverse immune pathways, functions, and cell types [27]. Based on the ssGSEA method, the NOTCH4-MUT tumors were characterized by more immune cells, such as TILs and CD8 T cells (P < 0.001, Fig. 4F). In addition, based on MCP-counter method, the NOTCH4-MUT tumors also tended to have more T cells, CD8 T cells, and cytotoxic lymphocytes (P < 0.001, Fig. 4G). Furthermore, the immune signatures (Fig. 5A) and microenvironment cell population (Fig. 5B) were obviously enriched in NOTCH4-MUT tumors compared to NOTCH4-WT tumors. According to the above results, the NOTCH4-MUT tumors had abundant immune cells.

When chemokines are combined with specific receptors, immune cells can accumulate in the TME [42]. We found that NOTCH4-MUT tumors had higher expression of immunostimulatory chemokines, including CXCL10 and CXCL9, which have been proven to attract CD8 T cells and dendritic cells (DCs) (Fig. 5C) [43, 44]. The higher expression of chemokines in NOTCH4-MUT tumors was compatible with the higher infiltration of immune cells in NOTCH4-MUT tumors, suggesting that NOTCH4-WT tumors were able to attract immune cells, which led to an extrinsic immune response.

Nonsilent somatic mutations in the coding region of a gene can generate presentable neoantigens, which can be recognized by T cells with structurally divergent antigen-specific TCRs [45]. We found significantly higher TCR diversity in NOTCH4-MUT tumors than in NOTCH4-WT tumors (P<0.001, Fig. 5D, E). After antigen stimulation-induced activation, T cells expand in the tumor to create an effector pool to perform their cytolytic function. Therefore, the cytolytic activity (CYT) score was determined to investigate the interplay between immune system activation and tumors. We found that NOTCH4-WT tumors were associated with significantly higher CYT scores (P < 0.001, Fig. 5F). From the above results, we can conclude that the NOTCH4-MUT tumors have more immune cells and higher TCR diversity to recognize tumor antigens and cause stronger tumor-killing effects.

## Discussion

The NOTCH pathway is an attractive cancer therapeutic target [4]. Numerous studies have suggested that Notch receptor family members play crucial roles in regulating a series of cellular processes, including progression and tumorigenesis, suggesting that these factors might serve as biomarkers for the prognosis and diagnosis of cancer [4]. However, their roles in cancer immunity have not been fully studied. In this study, based on carefully curated and collected response and genomic data, we investigate the relationship between NOTCH4 and the response to ICI therapy in an ICI-treated cohort. We observed that NOTCH4-MUT was enriched in patients responding to ICI therapy and strongly predicted clinical benefit. It was also found that NOTCH4-MUT was an independent and specific prognostic predictor for ICI therapy.

Moreover, we utilized the multidimensional TCGA dataset to dissect how NOTCH4-MUT tumors respond to immunotherapy. We found that NOTCH4-MUT tumors featured stronger immunogenicity, such as a higher TMB, and an inflammatory pattern of immune activities, such as high levels of CD8 T cell infiltration determined by the ESTIMATE method. When we used the ssGSEA method and MCP-counter method to calculate the overall immune cell infiltration levels in tumors, the immune score was found to be significantly higher in the NOTCH4-MUT tumors than in the NOTCH4-WT tumors, which again confirmed the elevated antitumor immune activity in the NOTCH4-MUT tumors. In fact, a large number of studies have indicated that the density of TILs is positively related to the immune response in various kinds of tumors [1]. In addition to a high degree of cytotoxic T cell infiltration, NOTCH4-MUT tumors were characterized by the overexpression of immune checkpoint factors, such as PD-1, PD-L1, and CTLA4, compared with NOTCH4-WT tumors. Therefore, enhanced tumor immunogenicity, activated antitumor immunity, and elevated PD-1, PD-L1, and CTLA4 expression could explain why NOTCH4-MUT tumors are more likely than NOTCH4-WT tumors to benefit from immunotherapy.

PD-L1 expression and the TMB were previously shown to be relevant to clinical benefit in patients treated with ICI therapy [1]. However, these two biomarkers are continuous variables without clearly defined cutoff points above which a response is guaranteed and below which a response does not occur. In addition, PD-L1 expression and the TMB both vary widely among different detection methods and platforms [46, 47]. In contrast, NOTCH4 mutations are easily detected by next-generation sequencing assays, and their presence in this study was strongly related to the response to ICI therapy. Therefore, it is worth considering a prospective basket trial that incorporates NOTCH4-MUT as a biomarker.

There are several limitations of this retrospective analysis. First, the ICI-treated cohort included patients receiving combination therapy based on anti-PD-1 and anti-PD-L1 antibodies. Different treatments can cause different responses. In addition, despite the strong correlation between NOTCH4-MUT and improved tumor immunogenicity as well as inflamed antitumor immunity, it is still necessary to further explore the potential molecular mechanism by which NOTCH4-MUT sensitizes patients to ICI therapy.

## Conclusions

In summary, the current study is the first to suggest that NOTCH4-MUT is related to strengthened tumor immunogenicity and inflamed antitumor immunity, which led to a better response and prolonged OS in cancer patients treated with ICI therapy, indicating that NOTCH4-MUT could be considered a potential predictive biomarker for ICI therapy. In the future, it is necessary to study the exact molecular mechanism, and large-scale prospective studies are also warranted.

## Supplementary information

**Additional file 1.** Table S1. Survival analysis was performed with 61 NOTCH4-MUT patients and 61 randomly selected NOTCH4-WT patients from 586 NOTCH4-WT patients, 1,000 samplings.

**Additional file 2.** Figure S1. Relationships among NOTCH4 status, PD-L1 status and TMB. (A) Predictive value of NOTCH4 mutation for the overall survival (OS) of NSCLC patients in the discovery cohort. (B) Predictive value of PD-L1 for the OS of NSCLC patients in the discovery cohort. (C) Predictive value of TMB for the OS of NSCLC patients in the discovery cohort. (D) Histogram showing the proportions of patients who achieved an objective response rate (ORR) among NOTCH4 MUT/PD-L1-negative/TMB-low and NOTCH4 MUT/PD-L1-positive/TMB-high patients. (E) Histogram showing the C-index of NOTCH4 status, PD-L1 status and TMB.

**Additional file 3.** Figure S2. Relationships between NOTCH4 status and objective response rate in lung adenocarcinoma and lung squamous cell carcinoma. (A) Histogram showing the proportions of patients who achieved an objective response rate (ORR) among NOTCH4-WT and NOTCH4-MUT patients with lung adenocarcinoma. (B) Histogram showing the proportions of patients who achieved an ORR among NOTCH4-WT and NOTCH4-MUT patients with lung squamous cell carcinoma. (C) Venn diagram showing the overlap between homologous repair gene mutations and NOTCH4 mutation in the discovery cohort. (D) Predictive value of NOTCH4 family mutation for overall survival (OS) in the discovery cohort. (E) Comparison of the NOTCH pathway score between NOTCH4-WT and NOTCH4-MUT in patients with lung adenocarcinoma in the TCGA dataset.

**Additional file 4.** Table S2. The activated and repressed genes in the NOTCH pathway.

**Additional file 5.** Figure S3. Relationships between NOTCH4 status and other characteristics. (A) Univariate and multivariate analyses of progression-free survival (PFS) according to NOTCH4 status. (B) Univariate and multivariate analyses of overall survival (OS) according to NOTCH4 status.

**Additional file 6.** Figure S4. Association of NOTCH4 status with prognosis in the validation cohort and non-ICI-treated cohort. (A) Predictive value of NOTCH4 mutation for overall survival (OS) in the validation cohort. (B) Predictive value of NOTCH4 mutation for OS in the non-ICI-treated cohort.

**Additional file 7.** Figure S5. Subgroup analyses of NOTCH4 status for each cancer type in the discovery cohort and in the validation cohort. (A) Predictive value of NOTCH4 mutation for overall survival (OS) in patients with non-small cell lung cancer in the discovery cohort. (B) Predictive value of NOTCH4 mutation for OS in patients with melanoma in the discovery cohort. (C) Predictive value of NOTCH4 mutation for OS in patients with non-small cell lung cancer in the validation cohort. (D) Predictive value of NOTCH4 mutation for OS in patients with melanoma in the validation cohort. (E) Predictive value of NOTCH4 mutation for OS in patients with renal cell carcinoma in the validation cohort. (F) Predictive value of NOTCH4 mutation for OS in patients with colorectal cancer in the validation cohort. (G) Predictive value of NOTCH4 mutation for OS in patients with esophagogastric cancer in the validation cohort. (H) Predictive value of NOTCH4 mutation for OS in patients with glioma in the validation cohort. (I) Predictive value of NOTCH4 mutation for OS in patients with bladder cancer in the validation cohort. (J) Predictive value of NOTCH4 mutation for OS in patients with head and neck cancer in the validation cohort. (K) Predictive value of NOTCH4 mutation for OS in patients with cancer of unknown origin in the validation cohort.

## Data Availability

All of the data we used in this study were publicly available in cBioPortal (https://www.cbioportal.org) and the PanCancer Atlas consortium (https://gdc.cancer.gov/about-data/publications/pancanatlas).

## Abbreviations

APM: Antigen-processing machinery
CNN: Convolutional neural network
CR: Complete response
CTLA-4: Cytotoxic T lymphocyte antigen 4
CYT: Cytolytic activity score
DC: Dendritic cell
DCB: Durable clinical benefit
FDA: Food and Drug Administration
GZMA: Granzyme A
H&E-stained: Hematoxylin and eosin-stained
HNSCC: Head and neck squamous cell carcinoma
HR: Hazard ratio
ICI: Immune checkpoint inhibitor
MCP: Microenvironment cell population
MHC: Major histocompatibility complex
MSK-IMPACT: Memorial Sloan Kettering-Integrated Mutation Profiling of Actionable Cancer Targets
MT: Mutant
NDB: No durable benefit
NOTCH4: NOTCH homolog-4
NSCLC: Non-small cell lung cancer
ORR: Objective response rate
OS: Overall survival
PD: Progression of disease
PD-(L)1: Programmed cell death (ligand) 1
PFS: Progression-free survival
PR: Partial response
PRF1: Perforin 1
RECIST: Response Evaluation Criteria in Solid Tumors
SD: Stable disease
ssGSEA: Single-sample gene set enrichment analysis
TCGA: The Cancer Genome Atlas
TCR: T cell receptor
TIL: Tumor-infiltrating lymphocyte
TMB: Tumor mutational burden
TME: Tumor microenvironment
WES: Whole-exome sequencing
WT: Wildtype
