# Supplementary material for: Identification of NOTCH4 mutation as a response biomarker for immune checkpoint inhibitor therapy
Source: BMC Med. 2021 Jul 21;19:154. doi: 10.1186/s12916-021-02031-3 (PMC8293505; doi:10.1186/s12916-021-02031-3)
Supplement: Supplementary file 1 — Additional file 1. Table S1. Survival analysis was performed with 61 NOTCH4-MUT patients and 61 randomly selected NOTCH4-WT patients from 586 NOTCH4-WT patients, 1,000 samplings. [file 12916_2021_2031_MOESM1_ESM.docx]

**Table S1. Survival analysis was performed with 61 NOTCH4-MUT patients and 61 NOTCH4-WT patients randomly selected from 586 NOTCH4-WT patients, with 1,000 samplings.**

| **Seed** | **The total number of NOTCH4-WT patients** | **The total number of NOTCH4-MUT patients** | **Median PFS of NOTCH4-WT patients** | **Median PFS of NOTCH4-MUT patients** | **P value (log-rank test)** | **Hazard ratio** |
| --- | --- | --- | --- | --- | --- | --- |
| 1 | 61 | 61 | 3.19 | 13.17 | 0.016793447 | 0.583150039 |
| 2 | 61 | 61 | 4.8 | 13.17 | 0.004965468 | 0.539931707 |
| 3 | 61 | 61 | 5.47 | 13.17 | 0.045764922 | 0.639995289 |
| 4 | 61 | 61 | 3.17 | 13.17 | 0.003765193 | 0.523780234 |
| 5 | 61 | 61 | 3.5 | 13.17 | 0.012073355 | 0.566729487 |
| 6 | 61 | 61 | 3.23 | 13.17 | 9.36E-05 | 0.426362449 |
| 7 | 61 | 61 | 3.5 | 13.17 | 0.002253765 | 0.508980534 |
| 8 | 61 | 61 | 4.51 | 13.17 | 0.014358851 | 0.581962337 |
| 9 | 61 | 61 | 3.133333333 | 13.17 | 0.000147447 | 0.426299276 |
| 10 | 61 | 61 | 4.433333333 | 13.17 | 0.000796382 | 0.475774015 |
| 11 | 61 | 61 | 3.17 | 13.17 | 0.000454136 | 0.471234449 |
| 12 | 61 | 61 | 4.43 | 13.17 | 0.024946904 | 0.60078554 |
| 13 | 61 | 61 | 3.266666667 | 13.17 | 0.000119074 | 0.427663277 |
| 14 | 61 | 61 | 3.6 | 13.17 | 0.002509596 | 0.499955096 |
| 15 | 61 | 61 | 3.91 | 13.17 | 0.021817458 | 0.593133319 |
| 16 | 61 | 61 | 3.6 | 13.17 | 0.007521784 | 0.547706174 |
| 17 | 61 | 61 | 3.5 | 13.17 | 0.006490789 | 0.548598784 |
| 18 | 61 | 61 | 3.26 | 13.17 | 0.014091489 | 0.57025484 |
| 19 | 61 | 61 | 3.75 | 13.17 | 0.050432507 | 0.638392436 |
| 20 | 61 | 61 | 4.27 | 13.17 | 0.006171226 | 0.539327146 |
| 21 | 61 | 61 | 3.17 | 13.17 | 0.000586342 | 0.474326329 |
| 22 | 61 | 61 | 3.19 | 13.17 | 0.000142975 | 0.435769488 |
| 23 | 61 | 61 | 5.37 | 13.17 | 0.288921497 | 0.781637279 |
| 24 | 61 | 61 | 4.2 | 13.17 | 0.010157479 | 0.564138449 |
| 25 | 61 | 61 | 3.22 | 13.17 | 0.026730144 | 0.602822476 |
| 26 | 61 | 61 | 4.24 | 13.17 | 0.004418071 | 0.528811564 |
| 27 | 61 | 61 | 3.45 | 13.17 | 0.001554778 | 0.500766582 |
| 28 | 61 | 61 | 3.033333333 | 13.17 | 0.006310217 | 0.543352137 |
| 29 | 61 | 61 | 5.53 | 13.17 | 0.073194644 | 0.664804941 |
| 30 | 61 | 61 | 4.57 | 13.17 | 0.004391331 | 0.538408463 |
| 31 | 61 | 61 | 4.2 | 13.17 | 0.009146868 | 0.555334648 |
| 32 | 61 | 61 | 3.033333333 | 13.17 | 0.001241378 | 0.488034979 |
| 33 | 61 | 61 | 2.96 | 13.17 | 2.57E-05 | 0.397924032 |
| 34 | 61 | 61 | 2.9 | 13.17 | 0.000110919 | 0.432296437 |
| 35 | 61 | 61 | 3.95 | 13.17 | 0.06767612 | 0.657100393 |
| 36 | 61 | 61 | 3.5 | 13.17 | 0.007628773 | 0.554269617 |
| 37 | 61 | 61 | 4.33 | 13.17 | 0.235306049 | 0.761353048 |
| 38 | 61 | 61 | 3.166666667 | 13.17 | 0.004016803 | 0.527586631 |
| 39 | 61 | 61 | 3.95 | 13.17 | 0.159361205 | 0.722898223 |
| 40 | 61 | 61 | 4.17 | 13.17 | 0.045615514 | 0.63476064 |
| 41 | 61 | 61 | 3.17 | 13.17 | 0.000610477 | 0.467900657 |
| 42 | 61 | 61 | 3.77 | 13.17 | 0.004859967 | 0.528412383 |
| 43 | 61 | 61 | 5.03 | 13.17 | 0.02819384 | 0.609696528 |
| 44 | 61 | 61 | 4.83 | 13.17 | 0.065496218 | 0.66002036 |
| 45 | 61 | 61 | 4.33 | 13.17 | 0.046747538 | 0.635523406 |
| 46 | 61 | 61 | 3.42 | 13.17 | 0.024229043 | 0.601946415 |
| 47 | 61 | 61 | 3.6 | 13.17 | 0.003199284 | 0.520537972 |
| 48 | 61 | 61 | 4.17 | 13.17 | 0.037705519 | 0.626200989 |
| 49 | 61 | 61 | 4.43 | 13.17 | 0.052272924 | 0.646154855 |
| 50 | 61 | 61 | 3.22 | 13.17 | 0.002361677 | 0.512907141 |
| 51 | 61 | 61 | 4.2 | 13.17 | 0.006883834 | 0.541181962 |
| 52 | 61 | 61 | 3.26 | 13.17 | 0.000149199 | 0.434872244 |
| 53 | 61 | 61 | 4 | 13.17 | 0.001704595 | 0.502368942 |
| 54 | 61 | 61 | 2.833333333 | 13.17 | 0.005107325 | 0.539728814 |
| 55 | 61 | 61 | 4.27 | 13.17 | 0.079035895 | 0.672046433 |
| 56 | 61 | 61 | 3.16 | 13.17 | 0.000808959 | 0.474610469 |
| 57 | 61 | 61 | 3.3 | 13.17 | 0.005460481 | 0.541644163 |
| 58 | 61 | 61 | 3.133333333 | 13.17 | 0.009061443 | 0.557054661 |
| 59 | 61 | 61 | 4.05 | 13.17 | 0.058646109 | 0.648755794 |
| 60 | 61 | 61 | 3.033333333 | 13.17 | 0.000276129 | 0.453243513 |
| 61 | 61 | 61 | 4.63 | 13.17 | 0.018770114 | 0.591555278 |
| 62 | 61 | 61 | 4.43 | 13.17 | 0.014205001 | 0.577447424 |
| 63 | 61 | 61 | 4.37 | 13.17 | 0.0142688 | 0.577502517 |
| 64 | 61 | 61 | 3.91 | 13.17 | 0.064667826 | 0.654589662 |
| 65 | 61 | 61 | 3.77 | 13.17 | 0.000228671 | 0.447909777 |
| 66 | 61 | 61 | 3.1 | 13.17 | 0.020699128 | 0.592647034 |
| 67 | 61 | 61 | 3.22 | 13.17 | 0.068612024 | 0.658887878 |
| 68 | 61 | 61 | 4.05 | 13.17 | 0.046786935 | 0.637052015 |
| 69 | 61 | 61 | 3.6 | 13.17 | 0.014157 | 0.576092636 |
| 70 | 61 | 61 | 4.97 | 13.17 | 0.114861379 | 0.699179568 |
| 71 | 61 | 61 | 3.333333333 | 13.17 | 0.00090979 | 0.487569474 |
| 72 | 61 | 61 | 3.23 | 13.17 | 0.006988249 | 0.548739531 |
| 73 | 61 | 61 | 4.4 | 13.17 | 0.019775524 | 0.59497106 |
| 74 | 61 | 61 | 3.6 | 13.17 | 0.005842794 | 0.544843279 |
| 75 | 61 | 61 | 4.27 | 13.17 | 0.09782362 | 0.685354416 |
| 76 | 61 | 61 | 3.42 | 13.17 | 0.002785564 | 0.51790911 |
| 77 | 61 | 61 | 3.65 | 13.17 | 0.019580193 | 0.594500928 |
| 78 | 61 | 61 | 3.6 | 13.17 | 0.00579873 | 0.541318123 |
| 79 | 61 | 61 | 4.24 | 13.17 | 0.024744464 | 0.604516039 |
| 80 | 61 | 61 | 3.39 | 13.17 | 0.002938437 | 0.521817816 |
| 81 | 61 | 61 | 3.42 | 13.17 | 0.01259682 | 0.573296695 |
| 82 | 61 | 61 | 2.966666667 | 13.17 | 0.004588564 | 0.532970737 |
| 83 | 61 | 61 | 2.66 | 13.17 | 0.00011734 | 0.432701607 |
| 84 | 61 | 61 | 3.26 | 13.17 | 0.004276776 | 0.530285833 |
| 85 | 61 | 61 | 3.91 | 13.17 | 0.019403276 | 0.586062065 |
| 86 | 61 | 61 | 4.17 | 13.17 | 0.008952849 | 0.559795424 |
| 87 | 61 | 61 | 3.91 | 13.17 | 0.000282004 | 0.452908245 |
| 88 | 61 | 61 | 4.2 | 13.17 | 0.010505115 | 0.566548378 |
| 89 | 61 | 61 | 3.45 | 13.17 | 0.000897077 | 0.479601448 |
| 90 | 61 | 61 | 2.966666667 | 13.17 | 0.000272545 | 0.450274952 |
| 91 | 61 | 61 | 3.166666667 | 13.17 | 0.01277833 | 0.572619947 |
| 92 | 61 | 61 | 2.733333333 | 13.17 | 0.000853829 | 0.478773283 |
| 93 | 61 | 61 | 5.33 | 13.17 | 0.126540095 | 0.704101904 |
| 94 | 61 | 61 | 2.8 | 13.17 | 0.000713291 | 0.477197704 |
| 95 | 61 | 61 | 4.83 | 13.17 | 0.021065765 | 0.588565717 |
| 96 | 61 | 61 | 4.83 | 13.17 | 0.019572083 | 0.596987926 |
| 97 | 61 | 61 | 4.7 | 13.17 | 0.014051233 | 0.574977341 |
| 98 | 61 | 61 | 3.43 | 13.17 | 0.01069497 | 0.565954845 |
| 99 | 61 | 61 | 3.133333333 | 13.17 | 0.000392527 | 0.451286876 |
| 100 | 61 | 61 | 3.166666667 | 13.17 | 0.002275958 | 0.506282604 |
| 101 | 61 | 61 | 3.5 | 13.17 | 0.000712326 | 0.475363616 |
| 102 | 61 | 61 | 3.133333333 | 13.17 | 0.001526275 | 0.49781225 |
| 103 | 61 | 61 | 3.5 | 13.17 | 0.016579909 | 0.58512852 |
| 104 | 61 | 61 | 5.27 | 13.17 | 0.036690954 | 0.625684143 |
| 105 | 61 | 61 | 3.19 | 13.17 | 0.000297811 | 0.440273558 |
| 106 | 61 | 61 | 4.57 | 13.17 | 0.015899196 | 0.583864502 |
| 107 | 61 | 61 | 2.76 | 13.17 | 0.000115166 | 0.434720014 |
| 108 | 61 | 61 | 3.91 | 13.17 | 0.022036577 | 0.59062634 |
| 109 | 61 | 61 | 3.5 | 13.17 | 0.017531714 | 0.584089608 |
| 110 | 61 | 61 | 3.6 | 13.17 | 0.02233387 | 0.593446824 |
| 111 | 61 | 61 | 3.75 | 13.17 | 0.013745001 | 0.577924009 |
| 112 | 61 | 61 | 4.73 | 13.17 | 0.040318802 | 0.631457274 |
| 113 | 61 | 61 | 3.49 | 13.17 | 0.006385731 | 0.54415114 |
| 114 | 61 | 61 | 2.93 | 13.17 | 0.005988591 | 0.544884862 |
| 115 | 61 | 61 | 5.03 | 13.17 | 0.060087974 | 0.643724055 |
| 116 | 61 | 61 | 3.68 | 13.17 | 0.001451256 | 0.496424954 |
| 117 | 61 | 61 | 2.9 | 13.17 | 0.014859115 | 0.576913152 |
| 118 | 61 | 61 | 3.39 | 13.17 | 0.001792013 | 0.503949776 |
| 119 | 61 | 61 | 2.73 | 13.17 | 0.002361221 | 0.511563843 |
| 120 | 61 | 61 | 2.8 | 13.17 | 0.001195121 | 0.490905158 |
| 121 | 61 | 61 | 3.22 | 13.17 | 0.011986362 | 0.571127627 |
| 122 | 61 | 61 | 3.033333333 | 13.17 | 0.000609443 | 0.466514545 |
| 123 | 61 | 61 | 4.57 | 13.17 | 0.018101962 | 0.593899355 |
| 124 | 61 | 61 | 2.76 | 13.17 | 0.000118234 | 0.432559301 |
| 125 | 61 | 61 | 4.17 | 13.17 | 0.011114328 | 0.563863607 |
| 126 | 61 | 61 | 3.49 | 13.17 | 0.005719648 | 0.534744729 |
| 127 | 61 | 61 | 3.42 | 13.17 | 0.011711215 | 0.573258808 |
| 128 | 61 | 61 | 2.966666667 | 13.17 | 0.002571684 | 0.518670063 |
| 129 | 61 | 61 | 3.6 | 13.17 | 0.029170832 | 0.602106068 |
| 130 | 61 | 61 | 4.43 | 13.17 | 0.00507545 | 0.535199369 |
| 131 | 61 | 61 | 2.93 | 13.17 | 0.000816523 | 0.478028657 |
| 132 | 61 | 61 | 2.833333333 | 13.17 | 6.45E-05 | 0.422173298 |
| 133 | 61 | 61 | 4.17 | 13.17 | 0.012155061 | 0.574636879 |
| 134 | 61 | 61 | 2.73 | 13.17 | 0.003245484 | 0.520726921 |
| 135 | 61 | 61 | 2.866666667 | 13.17 | 0.001239901 | 0.486939813 |
| 136 | 61 | 61 | 3.68 | 13.17 | 0.018704562 | 0.589458103 |
| 137 | 61 | 61 | 3.43 | 13.17 | 0.000231059 | 0.435708927 |
| 138 | 61 | 61 | 3.23 | 13.17 | 0.022963554 | 0.602056596 |
| 139 | 61 | 61 | 5.13 | 13.17 | 0.021754388 | 0.593474377 |
| 140 | 61 | 61 | 5.43 | 13.17 | 0.161121967 | 0.722050983 |
| 141 | 61 | 61 | 4.17 | 13.17 | 0.039029604 | 0.623759823 |
| 142 | 61 | 61 | 4.05 | 13.17 | 0.009668249 | 0.554450979 |
| 143 | 61 | 61 | 3.19 | 13.17 | 0.003286861 | 0.524918992 |
| 144 | 61 | 61 | 3.77 | 13.17 | 0.015682542 | 0.577250773 |
| 145 | 61 | 61 | 3.333333333 | 13.17 | 0.00442164 | 0.53160951 |
| 146 | 61 | 61 | 3.3 | 13.17 | 0.003052027 | 0.517618071 |
| 147 | 61 | 61 | 2.966666667 | 13.17 | 0.005083819 | 0.541630628 |
| 148 | 61 | 61 | 3.77 | 13.17 | 0.006842284 | 0.549275784 |
| 149 | 61 | 61 | 2.9 | 13.17 | 0.002176864 | 0.508955223 |
| 150 | 61 | 61 | 3.95 | 13.17 | 0.00603997 | 0.547993042 |
| 151 | 61 | 61 | 3.1 | 13.17 | 0.000698613 | 0.47495182 |
| 152 | 61 | 61 | 4.83 | 13.17 | 0.006285783 | 0.545889182 |
| 153 | 61 | 61 | 3.1 | 13.17 | 0.000497737 | 0.455928144 |
| 154 | 61 | 61 | 2.8 | 13.17 | 3.79E-05 | 0.401938305 |
| 155 | 61 | 61 | 3.43 | 13.17 | 0.007625164 | 0.542979043 |
| 156 | 61 | 61 | 3.39 | 13.17 | 0.002690878 | 0.519612738 |
| 157 | 61 | 61 | 3.6 | 13.17 | 0.012940021 | 0.56861846 |
| 158 | 61 | 61 | 4.43 | 13.17 | 0.007393381 | 0.555198038 |
| 159 | 61 | 61 | 4.24 | 13.17 | 0.159459805 | 0.723044949 |
| 160 | 61 | 61 | 3.45 | 13.17 | 0.05092612 | 0.641610814 |
| 161 | 61 | 61 | 3.19 | 13.17 | 0.00411272 | 0.522018652 |
| 162 | 61 | 61 | 4.24 | 13.17 | 0.028269717 | 0.607136623 |
| 163 | 61 | 61 | 4.33 | 13.17 | 0.016242372 | 0.588287834 |
| 164 | 61 | 61 | 4.54 | 13.17 | 0.167807711 | 0.727394646 |
| 165 | 61 | 61 | 3.91 | 13.17 | 0.005236478 | 0.536020422 |
| 166 | 61 | 61 | 4.4 | 13.17 | 0.008733331 | 0.563123728 |
| 167 | 61 | 61 | 3.65 | 13.17 | 0.008498848 | 0.558584875 |
| 168 | 61 | 61 | 3.07 | 13.17 | 0.016558467 | 0.585818066 |
| 169 | 61 | 61 | 3.17 | 13.17 | 0.004999027 | 0.529955272 |
| 170 | 61 | 61 | 3.5 | 13.17 | 0.018023482 | 0.59212652 |
| 171 | 61 | 61 | 3.03 | 13.17 | 0.00664252 | 0.548338977 |
| 172 | 61 | 61 | 2.8 | 13.17 | 0.000124284 | 0.434035938 |
| 173 | 61 | 61 | 2.8 | 13.17 | 0.001528172 | 0.496890064 |
| 174 | 61 | 61 | 3.333333333 | 13.17 | 0.006997965 | 0.547474062 |
| 175 | 61 | 61 | 2.9 | 13.17 | 0.000114226 | 0.42814712 |
| 176 | 61 | 61 | 4.37 | 13.17 | 0.004098578 | 0.52869725 |
| 177 | 61 | 61 | 3.52 | 13.17 | 0.011035288 | 0.566878772 |
| 178 | 61 | 61 | 3.95 | 13.17 | 0.056265316 | 0.647797132 |
| 179 | 61 | 61 | 3.5 | 13.17 | 0.014272276 | 0.575556661 |
| 180 | 61 | 61 | 3.1 | 13.17 | 0.000142686 | 0.432364267 |
| 181 | 61 | 61 | 3.333333333 | 13.17 | 0.003792696 | 0.531017446 |
| 182 | 61 | 61 | 3.17 | 13.17 | 0.003946665 | 0.529734074 |
| 183 | 61 | 61 | 3.65 | 13.17 | 0.021806725 | 0.593558499 |
| 184 | 61 | 61 | 3.033333333 | 13.17 | 0.000171152 | 0.441812213 |
| 185 | 61 | 61 | 5.13 | 13.17 | 0.133828745 | 0.710363836 |
| 186 | 61 | 61 | 3.91 | 13.17 | 0.013643351 | 0.576339937 |
| 187 | 61 | 61 | 4.37 | 13.17 | 0.010161099 | 0.556378691 |
| 188 | 61 | 61 | 4.17 | 13.17 | 0.016901908 | 0.586541617 |
| 189 | 61 | 61 | 4.27 | 13.17 | 0.017618819 | 0.590667055 |
| 190 | 61 | 61 | 3.91 | 13.17 | 0.075418375 | 0.664787905 |
| 191 | 61 | 61 | 3.166666667 | 13.17 | 0.002579916 | 0.514070448 |
| 192 | 61 | 61 | 5.4 | 13.17 | 0.022159237 | 0.60184856 |
| 193 | 61 | 61 | 2.8 | 13.17 | 0.002587606 | 0.511399993 |
| 194 | 61 | 61 | 3.43 | 13.17 | 0.005932377 | 0.542731347 |
| 195 | 61 | 61 | 3.133333333 | 13.17 | 0.003947987 | 0.531778656 |
| 196 | 61 | 61 | 2.93 | 13.17 | 1.13E-05 | 0.384182891 |
| 197 | 61 | 61 | 3.1 | 13.17 | 0.000545326 | 0.469217082 |
| 198 | 61 | 61 | 5.59 | 13.17 | 0.182586036 | 0.737211298 |
| 199 | 61 | 61 | 3.5 | 13.17 | 0.093190706 | 0.679410374 |
| 200 | 61 | 61 | 4.14 | 13.17 | 0.047944022 | 0.642613363 |
| 201 | 61 | 61 | 4.57 | 13.17 | 0.052357904 | 0.644409411 |
| 202 | 61 | 61 | 3.75 | 13.17 | 0.033938142 | 0.620941731 |
| 203 | 61 | 61 | 5.43 | 13.17 | 0.178296486 | 0.731352099 |
| 204 | 61 | 61 | 6.12 | 13.17 | 0.143009253 | 0.715807883 |
| 205 | 61 | 61 | 3.75 | 13.17 | 0.040638887 | 0.627592335 |
| 206 | 61 | 61 | 3.29 | 13.17 | 0.006285746 | 0.546182192 |
| 207 | 61 | 61 | 3.17 | 13.17 | 0.014869926 | 0.577671573 |
| 208 | 61 | 61 | 5.03 | 13.17 | 0.142994033 | 0.716145806 |
| 209 | 61 | 61 | 3.5 | 13.17 | 0.006438051 | 0.544707743 |
| 210 | 61 | 61 | 3.033333333 | 13.17 | 0.009786833 | 0.566194621 |
| 211 | 61 | 61 | 3.5 | 13.17 | 0.003754953 | 0.530726728 |
| 212 | 61 | 61 | 3.8 | 13.17 | 0.005644852 | 0.540179213 |
| 213 | 61 | 61 | 2.766666667 | 13.17 | 0.000127993 | 0.429069893 |
| 214 | 61 | 61 | 2.833333333 | 13.17 | 0.000621352 | 0.477207484 |
| 215 | 61 | 61 | 4.2 | 13.17 | 0.002865231 | 0.518888354 |
| 216 | 61 | 61 | 6.57 | 13.17 | 0.19137063 | 0.739923648 |
| 217 | 61 | 61 | 4.27 | 13.17 | 0.141067841 | 0.712463894 |
| 218 | 61 | 61 | 3.333333333 | 13.17 | 0.000140595 | 0.444745151 |
| 219 | 61 | 61 | 4 | 13.17 | 0.028007513 | 0.609359822 |
| 220 | 61 | 61 | 3.5 | 13.17 | 0.001340176 | 0.492852202 |
| 221 | 61 | 61 | 4.27 | 13.17 | 0.002211684 | 0.513067926 |
| 222 | 61 | 61 | 4.14 | 13.17 | 0.032897167 | 0.610333664 |
| 223 | 61 | 61 | 6.3 | 13.17 | 0.077500774 | 0.667704781 |
| 224 | 61 | 61 | 4.11 | 13.17 | 0.127488035 | 0.707651783 |
| 225 | 61 | 61 | 4.97 | 13.17 | 0.07029267 | 0.663229249 |
| 226 | 61 | 61 | 3.166666667 | 13.17 | 0.003052645 | 0.523321325 |
| 227 | 61 | 61 | 3.29 | 13.17 | 0.012070557 | 0.564977362 |
| 228 | 61 | 61 | 4.37 | 13.17 | 0.005256327 | 0.537092027 |
| 229 | 61 | 61 | 5.53 | 13.17 | 0.086701838 | 0.677358942 |
| 230 | 61 | 61 | 3.3 | 13.17 | 0.012477054 | 0.57241133 |
| 231 | 61 | 61 | 3.29 | 13.17 | 0.003574148 | 0.52640525 |
| 232 | 61 | 61 | 6.13 | 13.17 | 0.102137146 | 0.689285278 |
| 233 | 61 | 61 | 3.5 | 13.17 | 0.003851011 | 0.524766515 |
| 234 | 61 | 61 | 5.1 | 13.17 | 0.070248837 | 0.661594327 |
| 235 | 61 | 61 | 3.17 | 13.17 | 0.000148224 | 0.431487426 |
| 236 | 61 | 61 | 4.8 | 13.17 | 0.006982256 | 0.554842573 |
| 237 | 61 | 61 | 3.95 | 13.17 | 0.027046024 | 0.613893588 |
| 238 | 61 | 61 | 2.76 | 13.17 | 0.00107069 | 0.482178012 |
| 239 | 61 | 61 | 3.29 | 13.17 | 0.000984281 | 0.483366124 |
| 240 | 61 | 61 | 3.43 | 13.17 | 0.027234928 | 0.609382597 |
| 241 | 61 | 61 | 2.76 | 13.17 | 3.38E-05 | 0.414078817 |
| 242 | 61 | 61 | 3.07 | 13.17 | 0.013776034 | 0.573829734 |
| 243 | 61 | 61 | 4.133333333 | 13.17 | 0.023151497 | 0.603646898 |
| 244 | 61 | 61 | 3.65 | 13.17 | 0.003544786 | 0.521868355 |
| 245 | 61 | 61 | 3.5 | 13.17 | 0.005491314 | 0.543795667 |
| 246 | 61 | 61 | 4.43 | 13.17 | 0.077130563 | 0.671434089 |
| 247 | 61 | 61 | 4.27 | 13.17 | 0.049250241 | 0.644099231 |
| 248 | 61 | 61 | 5.43 | 13.17 | 0.013434595 | 0.570889518 |
| 249 | 61 | 61 | 3.133333333 | 13.17 | 0.011652081 | 0.566361667 |
| 250 | 61 | 61 | 3.43 | 13.17 | 0.004719381 | 0.536960935 |
| 251 | 61 | 61 | 4.37 | 13.17 | 0.004585177 | 0.526953603 |
| 252 | 61 | 61 | 3.8 | 13.17 | 0.003490476 | 0.525572697 |
| 253 | 61 | 61 | 3.03 | 13.17 | 0.000581332 | 0.467990413 |
| 254 | 61 | 61 | 2.966666667 | 13.17 | 0.00334641 | 0.514532434 |
| 255 | 61 | 61 | 4.27 | 13.17 | 0.00862437 | 0.557308999 |
| 256 | 61 | 61 | 2.966666667 | 13.17 | 5.60E-05 | 0.417347358 |
| 257 | 61 | 61 | 3.22 | 13.17 | 0.072727218 | 0.6633583 |
| 258 | 61 | 61 | 2.8 | 13.17 | 0.006442503 | 0.544472679 |
| 259 | 61 | 61 | 4.41 | 13.17 | 0.008789196 | 0.5594036 |
| 260 | 61 | 61 | 4.54 | 13.17 | 0.005829934 | 0.54635702 |
| 261 | 61 | 61 | 3.17 | 13.17 | 0.000727902 | 0.478471113 |
| 262 | 61 | 61 | 6.033333333 | 13.17 | 0.124012631 | 0.703558648 |
| 263 | 61 | 61 | 3.65 | 13.17 | 0.005724398 | 0.540871028 |
| 264 | 61 | 61 | 3.1 | 13.17 | 0.002115718 | 0.509892474 |
| 265 | 61 | 61 | 3.8 | 13.17 | 0.00501801 | 0.533034996 |
| 266 | 61 | 61 | 4.41 | 13.17 | 0.07107132 | 0.664388746 |
| 267 | 61 | 61 | 3.77 | 13.17 | 0.036081554 | 0.623255971 |
| 268 | 61 | 61 | 3.6 | 13.17 | 0.001308473 | 0.487246328 |
| 269 | 61 | 61 | 5.59 | 13.17 | 0.040598819 | 0.629323869 |
| 270 | 61 | 61 | 3.5 | 13.17 | 0.006978009 | 0.551656883 |
| 271 | 61 | 61 | 4.17 | 13.17 | 0.040942109 | 0.628331214 |
| 272 | 61 | 61 | 4.4 | 13.17 | 0.022006836 | 0.600270951 |
| 273 | 61 | 61 | 3.07 | 13.17 | 0.009521469 | 0.55927981 |
| 274 | 61 | 61 | 4.133333333 | 13.17 | 0.00949867 | 0.560008485 |
| 275 | 61 | 61 | 3.033333333 | 13.17 | 0.000703393 | 0.469189843 |
| 276 | 61 | 61 | 4 | 13.17 | 0.01682849 | 0.586480492 |
| 277 | 61 | 61 | 3.43 | 13.17 | 0.034892219 | 0.623330163 |
| 278 | 61 | 61 | 5.99 | 13.17 | 0.22200012 | 0.753693828 |
| 279 | 61 | 61 | 2.866666667 | 13.17 | 0.004005319 | 0.529003286 |
| 280 | 61 | 61 | 3.5 | 13.17 | 0.003316989 | 0.522981488 |
| 281 | 61 | 61 | 2.566666667 | 13.17 | 0.000131249 | 0.438387659 |
| 282 | 61 | 61 | 4.57 | 13.17 | 0.021771229 | 0.598129804 |
| 283 | 61 | 61 | 4.4 | 13.17 | 0.051256105 | 0.640109348 |
| 284 | 61 | 61 | 3.16 | 13.17 | 0.002293806 | 0.500919751 |
| 285 | 61 | 61 | 3.5 | 13.17 | 0.004824309 | 0.539591579 |
| 286 | 61 | 61 | 4.41 | 13.17 | 0.003305185 | 0.515599231 |
| 287 | 61 | 61 | 3.3 | 13.17 | 0.033748741 | 0.619946989 |
| 288 | 61 | 61 | 4.67 | 13.17 | 0.070173898 | 0.658848938 |
| 289 | 61 | 61 | 5.27 | 13.17 | 0.057826668 | 0.650300328 |
| 290 | 61 | 61 | 3.3 | 13.17 | 0.004881957 | 0.541690588 |
| 291 | 61 | 61 | 4.11 | 13.17 | 0.026139181 | 0.605017433 |
| 292 | 61 | 61 | 4.51 | 13.17 | 0.001405248 | 0.481929823 |
| 293 | 61 | 61 | 4.4 | 13.17 | 0.134309045 | 0.7085396 |
| 294 | 61 | 61 | 3.29 | 13.17 | 0.000222312 | 0.440996507 |
| 295 | 61 | 61 | 3.3 | 13.17 | 0.001092028 | 0.490918509 |
| 296 | 61 | 61 | 4.14 | 13.17 | 0.04427596 | 0.633595245 |
| 297 | 61 | 61 | 3.3 | 13.17 | 0.000257634 | 0.435270552 |
| 298 | 61 | 61 | 4.73 | 13.17 | 0.021240692 | 0.59598993 |
| 299 | 61 | 61 | 3.8 | 13.17 | 0.002572367 | 0.519170611 |
| 300 | 61 | 61 | 3.75 | 13.17 | 0.007583666 | 0.540173671 |
| 301 | 61 | 61 | 5.43 | 13.17 | 0.003881281 | 0.533025089 |
| 302 | 61 | 61 | 2.57 | 13.17 | 2.82E-05 | 0.399476424 |
| 303 | 61 | 61 | 3.266666667 | 13.17 | 0.008752751 | 0.557922195 |
| 304 | 61 | 61 | 3.266666667 | 13.17 | 0.00255821 | 0.515108189 |
| 305 | 61 | 61 | 2.73 | 13.17 | 0.001473279 | 0.496916325 |
| 306 | 61 | 61 | 3.95 | 13.17 | 0.004144384 | 0.524429748 |
| 307 | 61 | 61 | 7.2 | 13.17 | 0.090912985 | 0.677907454 |
| 308 | 61 | 61 | 3.266666667 | 13.17 | 0.003029376 | 0.517061331 |
| 309 | 61 | 61 | 4.73 | 13.17 | 0.077712462 | 0.670641369 |
| 310 | 61 | 61 | 3.75 | 13.17 | 0.035511969 | 0.622704788 |
| 311 | 61 | 61 | 4.17 | 13.17 | 0.025810828 | 0.606923583 |
| 312 | 61 | 61 | 3.95 | 13.17 | 0.008989324 | 0.555644319 |
| 313 | 61 | 61 | 5.37 | 13.17 | 0.127887673 | 0.706828373 |
| 314 | 61 | 61 | 4.7 | 13.17 | 0.102447973 | 0.686202949 |
| 315 | 61 | 61 | 4.133333333 | 13.17 | 0.029164822 | 0.607766605 |
| 316 | 61 | 61 | 3.95 | 13.17 | 0.002045912 | 0.505230617 |
| 317 | 61 | 61 | 2.8 | 13.17 | 0.005075997 | 0.53439742 |
| 318 | 61 | 61 | 3.6 | 13.17 | 0.008455868 | 0.560597738 |
| 319 | 61 | 61 | 3.65 | 13.17 | 0.009907685 | 0.563198815 |
| 320 | 61 | 61 | 4.24 | 13.17 | 0.014311062 | 0.573856259 |
| 321 | 61 | 61 | 3.5 | 13.17 | 0.002013092 | 0.508305451 |
| 322 | 61 | 61 | 6.333333333 | 13.17 | 0.200020842 | 0.74875161 |
| 323 | 61 | 61 | 2.866666667 | 13.17 | 0.000633039 | 0.465053099 |
| 324 | 61 | 61 | 3.52 | 13.17 | 0.003299602 | 0.523419508 |
| 325 | 61 | 61 | 2.8 | 13.17 | 0.004444996 | 0.535127475 |
| 326 | 61 | 61 | 3.17 | 13.17 | 0.000873142 | 0.477079751 |
| 327 | 61 | 61 | 3.8 | 13.17 | 0.011334446 | 0.571651555 |
| 328 | 61 | 61 | 5.77 | 13.17 | 0.132801443 | 0.706851545 |
| 329 | 61 | 61 | 2.53 | 13.17 | 1.57E-06 | 0.360569246 |
| 330 | 61 | 61 | 4.14 | 13.17 | 0.003029395 | 0.516208504 |
| 331 | 61 | 61 | 3.5 | 13.17 | 0.009066023 | 0.559336778 |
| 332 | 61 | 61 | 3.17 | 13.17 | 0.003715303 | 0.521363378 |
| 333 | 61 | 61 | 3.1 | 13.17 | 0.00237092 | 0.513285677 |
| 334 | 61 | 61 | 4.54 | 13.17 | 0.007002899 | 0.549738315 |
| 335 | 61 | 61 | 2.8 | 13.17 | 3.63E-05 | 0.405350348 |
| 336 | 61 | 61 | 3.65 | 13.17 | 0.003149427 | 0.523719962 |
| 337 | 61 | 61 | 5.37 | 13.17 | 0.036969048 | 0.623800036 |
| 338 | 61 | 61 | 3.91 | 13.17 | 0.006982721 | 0.552358936 |
| 339 | 61 | 61 | 5.53 | 13.17 | 0.236150373 | 0.760459534 |
| 340 | 61 | 61 | 4.11 | 13.17 | 0.002049416 | 0.501504037 |
| 341 | 61 | 61 | 3.266666667 | 13.17 | 0.000844208 | 0.481236635 |
| 342 | 61 | 61 | 4.14 | 13.17 | 0.004388748 | 0.528823731 |
| 343 | 61 | 61 | 5.13 | 13.17 | 0.087902702 | 0.67993308 |
| 344 | 61 | 61 | 3.23 | 13.17 | 0.009407746 | 0.561102498 |
| 345 | 61 | 61 | 4.83 | 13.17 | 0.07594483 | 0.668972115 |
| 346 | 61 | 61 | 3.91 | 13.17 | 0.007319885 | 0.545135859 |
| 347 | 61 | 61 | 5.4 | 13.17 | 0.15836509 | 0.724041853 |
| 348 | 61 | 61 | 2.833333333 | 13.17 | 0.000602034 | 0.470683022 |
| 349 | 61 | 61 | 4.27 | 13.17 | 0.008999617 | 0.558952228 |
| 350 | 61 | 61 | 4.67 | 13.17 | 0.030398129 | 0.6062237 |
| 351 | 61 | 61 | 2.76 | 13.17 | 4.23E-05 | 0.408646497 |
| 352 | 61 | 61 | 3.16 | 13.17 | 0.002175175 | 0.503132193 |
| 353 | 61 | 61 | 6.033333333 | 13.17 | 0.252192558 | 0.771596824 |
| 354 | 61 | 61 | 4.14 | 13.17 | 0.022837157 | 0.59923943 |
| 355 | 61 | 61 | 5.53 | 13.17 | 0.081814781 | 0.667159078 |
| 356 | 61 | 61 | 3.6 | 13.17 | 0.026249338 | 0.601044811 |
| 357 | 61 | 61 | 3.5 | 13.17 | 0.056803262 | 0.652996463 |
| 358 | 61 | 61 | 4.433333333 | 13.17 | 0.015203283 | 0.574875922 |
| 359 | 61 | 61 | 2.833333333 | 13.17 | 1.95E-05 | 0.390535757 |
| 360 | 61 | 61 | 3.133333333 | 13.17 | 0.001865931 | 0.500169731 |
| 361 | 61 | 61 | 5.37 | 13.17 | 0.286862985 | 0.777257741 |
| 362 | 61 | 61 | 3.133333333 | 13.17 | 0.003321879 | 0.527925522 |
| 363 | 61 | 61 | 4.11 | 13.17 | 0.01585367 | 0.585328269 |
| 364 | 61 | 61 | 3.8 | 13.17 | 0.026699871 | 0.61258069 |
| 365 | 61 | 61 | 4.33 | 13.17 | 0.016811214 | 0.589208605 |
| 366 | 61 | 61 | 3.033333333 | 13.17 | 0.010456361 | 0.5625028 |
| 367 | 61 | 61 | 3.17 | 13.17 | 0.000855751 | 0.477923242 |
| 368 | 61 | 61 | 4.2 | 13.17 | 0.006685915 | 0.548596792 |
| 369 | 61 | 61 | 2.8 | 13.17 | 0.000182424 | 0.441555243 |
| 370 | 61 | 61 | 4.43 | 13.17 | 0.048337209 | 0.641163119 |
| 371 | 61 | 61 | 5.03 | 13.17 | 0.116430435 | 0.696861816 |
| 372 | 61 | 61 | 4.73 | 13.17 | 0.067135307 | 0.661278544 |
| 373 | 61 | 61 | 3.91 | 13.17 | 0.013276439 | 0.580909613 |
| 374 | 61 | 61 | 2.833333333 | 13.17 | 0.00013609 | 0.438531728 |
| 375 | 61 | 61 | 2.96 | 13.17 | 0.000216683 | 0.438519383 |
| 376 | 61 | 61 | 4.63 | 13.17 | 0.035757665 | 0.621899056 |
| 377 | 61 | 61 | 2.8 | 13.17 | 0.000217183 | 0.446911055 |
| 378 | 61 | 61 | 4.11 | 13.17 | 0.002332738 | 0.507323721 |
| 379 | 61 | 61 | 3.5 | 13.17 | 0.006564333 | 0.544934566 |
| 380 | 61 | 61 | 3.23 | 13.17 | 0.006967631 | 0.546283329 |
| 381 | 61 | 61 | 3.3 | 13.17 | 0.000408302 | 0.460332565 |
| 382 | 61 | 61 | 3.26 | 13.17 | 0.011806087 | 0.573387224 |
| 383 | 61 | 61 | 3.6 | 13.17 | 0.008298132 | 0.54999498 |
| 384 | 61 | 61 | 4.11 | 13.17 | 0.010082467 | 0.562952695 |
| 385 | 61 | 61 | 2.866666667 | 13.17 | 0.002156894 | 0.505239708 |
| 386 | 61 | 61 | 5.47 | 13.17 | 0.005498489 | 0.542467246 |
| 387 | 61 | 61 | 3.65 | 13.17 | 0.00152107 | 0.499037112 |
| 388 | 61 | 61 | 3.91 | 13.17 | 0.052403094 | 0.642537742 |
| 389 | 61 | 61 | 2.8 | 13.17 | 0.00386626 | 0.529852224 |
| 390 | 61 | 61 | 3.03 | 13.17 | 0.000869474 | 0.481749361 |
| 391 | 61 | 61 | 4.133333333 | 13.17 | 0.039119247 | 0.624777584 |
| 392 | 61 | 61 | 3.5 | 13.17 | 0.001668279 | 0.504202196 |
| 393 | 61 | 61 | 3.5 | 13.17 | 0.000917837 | 0.475029479 |
| 394 | 61 | 61 | 4.17 | 13.17 | 0.039515582 | 0.626943872 |
| 395 | 61 | 61 | 3.26 | 13.17 | 0.0026106 | 0.51757738 |
| 396 | 61 | 61 | 4.27 | 13.17 | 0.026729826 | 0.610493512 |
| 397 | 61 | 61 | 4.14 | 13.17 | 0.029050127 | 0.609413486 |
| 398 | 61 | 61 | 3.75 | 13.17 | 0.020017411 | 0.594741902 |
| 399 | 61 | 61 | 3.5 | 13.17 | 0.000180323 | 0.428745751 |
| 400 | 61 | 61 | 2.8 | 13.17 | 7.76E-05 | 0.413914984 |
| 401 | 61 | 61 | 3.17 | 13.17 | 0.001919609 | 0.502084173 |
| 402 | 61 | 61 | 3.91 | 13.17 | 0.009247568 | 0.559683042 |
| 403 | 61 | 61 | 4.17 | 13.17 | 0.012610727 | 0.575743718 |
| 404 | 61 | 61 | 3.6 | 13.17 | 0.014111191 | 0.575673414 |
| 405 | 61 | 61 | 3.6 | 13.17 | 0.006043916 | 0.54789053 |
| 406 | 61 | 61 | 4.11 | 13.17 | 0.036801016 | 0.627316283 |
| 407 | 61 | 61 | 3.49 | 13.17 | 0.015179235 | 0.581385861 |
| 408 | 61 | 61 | 4.17 | 13.17 | 0.051968261 | 0.644417477 |
| 409 | 61 | 61 | 4.57 | 13.17 | 0.02334006 | 0.606624921 |
| 410 | 61 | 61 | 3.033333333 | 13.17 | 0.00552285 | 0.535260825 |
| 411 | 61 | 61 | 2.96 | 13.17 | 0.006318532 | 0.546476447 |
| 412 | 61 | 61 | 5.43 | 13.17 | 0.023913869 | 0.599861273 |
| 413 | 61 | 61 | 3.65 | 13.17 | 0.009997168 | 0.564275089 |
| 414 | 61 | 61 | 3.95 | 13.17 | 0.004512799 | 0.529627046 |
| 415 | 61 | 61 | 3.17 | 13.17 | 0.004861569 | 0.529421296 |
| 416 | 61 | 61 | 4.14 | 13.17 | 0.014620616 | 0.579327686 |
| 417 | 61 | 61 | 4.27 | 13.17 | 0.008713226 | 0.555928628 |
| 418 | 61 | 61 | 4.57 | 13.17 | 0.075453443 | 0.664927682 |
| 419 | 61 | 61 | 2.9 | 13.17 | 8.83E-05 | 0.421151837 |
| 420 | 61 | 61 | 4.433333333 | 13.17 | 0.028502082 | 0.614539058 |
| 421 | 61 | 61 | 3.95 | 13.17 | 0.057328912 | 0.650715996 |
| 422 | 61 | 61 | 3.39 | 13.17 | 0.00185973 | 0.501464402 |
| 423 | 61 | 61 | 3.26 | 13.17 | 0.003759807 | 0.5262384 |
| 424 | 61 | 61 | 3.5 | 13.17 | 0.056426212 | 0.646803989 |
| 425 | 61 | 61 | 3.19 | 13.17 | 0.019331664 | 0.597533226 |
| 426 | 61 | 61 | 4.7 | 13.17 | 0.032106666 | 0.609758928 |
| 427 | 61 | 61 | 3.5 | 13.17 | 0.006460894 | 0.54213313 |
| 428 | 61 | 61 | 3.26 | 13.17 | 0.009688224 | 0.562244335 |
| 429 | 61 | 61 | 3.26 | 13.17 | 0.006615407 | 0.543302337 |
| 430 | 61 | 61 | 3.95 | 13.17 | 0.001242359 | 0.48717887 |
| 431 | 61 | 61 | 3.6 | 13.17 | 0.000661288 | 0.466345542 |
| 432 | 61 | 61 | 3.6 | 13.17 | 0.009263533 | 0.556296249 |
| 433 | 61 | 61 | 2.833333333 | 13.17 | 0.001232743 | 0.493353477 |
| 434 | 61 | 61 | 3.5 | 13.17 | 0.046473381 | 0.632856203 |
| 435 | 61 | 61 | 4.41 | 13.17 | 0.035574449 | 0.623520846 |
| 436 | 61 | 61 | 3.43 | 13.17 | 0.003598854 | 0.527164455 |
| 437 | 61 | 61 | 7.8 | 13.17 | 0.355403669 | 0.805955976 |
| 438 | 61 | 61 | 4.2 | 13.17 | 0.007625161 | 0.550617837 |
| 439 | 61 | 61 | 4.7 | 13.17 | 0.010860723 | 0.565491252 |
| 440 | 61 | 61 | 4.37 | 13.17 | 0.001685171 | 0.496862574 |
| 441 | 61 | 61 | 4.133333333 | 13.17 | 0.003430731 | 0.524956257 |
| 442 | 61 | 61 | 3.266666667 | 13.17 | 0.001599271 | 0.503116687 |
| 443 | 61 | 61 | 3.033333333 | 13.17 | 0.005259292 | 0.540860023 |
| 444 | 61 | 61 | 3.75 | 13.17 | 0.008924966 | 0.555459813 |
| 445 | 61 | 61 | 4.133333333 | 13.17 | 0.008804571 | 0.554415842 |
| 446 | 61 | 61 | 2.733333333 | 13.17 | 0.0004334 | 0.468232586 |
| 447 | 61 | 61 | 3.42 | 13.17 | 0.004228767 | 0.534163269 |
| 448 | 61 | 61 | 5.03 | 13.17 | 0.040224409 | 0.62826829 |
| 449 | 61 | 61 | 2.76 | 13.17 | 5.96E-05 | 0.417813115 |
| 450 | 61 | 61 | 4.33 | 13.17 | 0.068175577 | 0.658207451 |
| 451 | 61 | 61 | 4.4 | 13.17 | 0.011757445 | 0.568096596 |
| 452 | 61 | 61 | 5.03 | 13.17 | 0.031007343 | 0.613649044 |
| 453 | 61 | 61 | 3.91 | 13.17 | 0.022975077 | 0.603064654 |
| 454 | 61 | 61 | 4.43 | 13.17 | 0.064578048 | 0.654964753 |
| 455 | 61 | 61 | 4.05 | 13.17 | 0.014373161 | 0.58395629 |
| 456 | 61 | 61 | 3.5 | 13.17 | 0.018239879 | 0.591431528 |
| 457 | 61 | 61 | 3.49 | 13.17 | 0.000720833 | 0.474889187 |
| 458 | 61 | 61 | 4.17 | 13.17 | 0.01359396 | 0.574272766 |
| 459 | 61 | 61 | 3.52 | 13.17 | 0.029863092 | 0.610509553 |
| 460 | 61 | 61 | 2.8 | 13.17 | 0.024380435 | 0.600287751 |
| 461 | 61 | 61 | 3.5 | 13.17 | 0.003856877 | 0.528351963 |
| 462 | 61 | 61 | 4.17 | 13.17 | 0.082797711 | 0.672323925 |
| 463 | 61 | 61 | 5.43 | 13.17 | 0.246631254 | 0.767218438 |
| 464 | 61 | 61 | 3.77 | 13.17 | 0.010062206 | 0.557565632 |
| 465 | 61 | 61 | 2.8 | 13.17 | 0.000482361 | 0.462488093 |
| 466 | 61 | 61 | 3.6 | 13.17 | 0.015219735 | 0.582060031 |
| 467 | 61 | 61 | 3.22 | 13.17 | 0.001110631 | 0.484295786 |
| 468 | 61 | 61 | 4.2 | 13.17 | 0.003667762 | 0.520919558 |
| 469 | 61 | 61 | 6.17 | 13.17 | 0.064436247 | 0.659156587 |
| 470 | 61 | 61 | 4.133333333 | 13.17 | 0.002525962 | 0.514729333 |
| 471 | 61 | 61 | 3.17 | 13.17 | 0.011239898 | 0.565091288 |
| 472 | 61 | 61 | 5.53 | 13.17 | 0.421698365 | 0.829557859 |
| 473 | 61 | 61 | 3.17 | 13.17 | 0.001582392 | 0.501588496 |
| 474 | 61 | 61 | 2.67 | 13.17 | 0.000122809 | 0.429510175 |
| 475 | 61 | 61 | 3.5 | 13.17 | 2.95E-05 | 0.400365213 |
| 476 | 61 | 61 | 4.57 | 13.17 | 0.170751198 | 0.73066694 |
| 477 | 61 | 61 | 2.966666667 | 13.17 | 0.001784959 | 0.501464779 |
| 478 | 61 | 61 | 3.75 | 13.17 | 0.023739455 | 0.606986825 |
| 479 | 61 | 61 | 3.91 | 13.17 | 0.005551508 | 0.535145629 |
| 480 | 61 | 61 | 3.95 | 13.17 | 0.004575457 | 0.52176197 |
| 481 | 61 | 61 | 5.99 | 13.17 | 0.138034408 | 0.712332752 |
| 482 | 61 | 61 | 3.07 | 13.17 | 0.001277321 | 0.499732242 |
| 483 | 61 | 61 | 4.133333333 | 13.17 | 0.011000189 | 0.56778569 |
| 484 | 61 | 61 | 3.5 | 13.17 | 0.012908239 | 0.576408726 |
| 485 | 61 | 61 | 3.42 | 13.17 | 0.004498277 | 0.529313006 |
| 486 | 61 | 61 | 4.83 | 13.17 | 0.005884291 | 0.532596415 |
| 487 | 61 | 61 | 2.966666667 | 13.17 | 0.000790575 | 0.478944392 |
| 488 | 61 | 61 | 3.6 | 13.17 | 0.004950391 | 0.536073999 |
| 489 | 61 | 61 | 3.91 | 13.17 | 6.91E-05 | 0.414710675 |
| 490 | 61 | 61 | 3.16 | 13.17 | 0.00232641 | 0.506935257 |
| 491 | 61 | 61 | 3.29 | 13.17 | 0.001095542 | 0.484453709 |
| 492 | 61 | 61 | 4.2 | 13.17 | 0.00401668 | 0.526653004 |
| 493 | 61 | 61 | 5.77 | 13.17 | 0.109970892 | 0.697012452 |
| 494 | 61 | 61 | 3.033333333 | 13.17 | 0.000250312 | 0.447125948 |
| 495 | 61 | 61 | 4.7 | 13.17 | 0.019332275 | 0.597560127 |
| 496 | 61 | 61 | 4.83 | 13.17 | 0.061979034 | 0.65878724 |
| 497 | 61 | 61 | 5.03 | 13.17 | 0.259522135 | 0.76853167 |
| 498 | 61 | 61 | 3.26 | 13.17 | 0.000752935 | 0.479222355 |
| 499 | 61 | 61 | 7.99 | 13.17 | 0.288548303 | 0.787042035 |
| 500 | 61 | 61 | 4.05 | 13.17 | 0.01423882 | 0.576546314 |
| 501 | 61 | 61 | 3.39 | 13.17 | 0.074557132 | 0.664479065 |
| 502 | 61 | 61 | 4.17 | 13.17 | 0.005112975 | 0.535936942 |
| 503 | 61 | 61 | 3.68 | 13.17 | 0.081424294 | 0.672333705 |
| 504 | 61 | 61 | 4.43 | 13.17 | 0.015041119 | 0.58048123 |
| 505 | 61 | 61 | 3.91 | 13.17 | 0.022743458 | 0.603399977 |
| 506 | 61 | 61 | 5.27 | 13.17 | 0.031532583 | 0.61157921 |
| 507 | 61 | 61 | 5.1 | 13.17 | 0.086910659 | 0.683612242 |
| 508 | 61 | 61 | 3.5 | 13.17 | 0.004584805 | 0.537751495 |
| 509 | 61 | 61 | 2.8 | 13.17 | 0.000485525 | 0.462884118 |
| 510 | 61 | 61 | 4.17 | 13.17 | 0.029780902 | 0.613780657 |
| 511 | 61 | 61 | 3.5 | 13.17 | 0.001018981 | 0.483605275 |
| 512 | 61 | 61 | 3.3 | 13.17 | 0.02690193 | 0.608706415 |
| 513 | 61 | 61 | 3.6 | 13.17 | 0.01457514 | 0.582510286 |
| 514 | 61 | 61 | 4.133333333 | 13.17 | 0.016118448 | 0.587578617 |
| 515 | 61 | 61 | 4.43 | 13.17 | 0.002041927 | 0.512461326 |
| 516 | 61 | 61 | 3.42 | 13.17 | 0.00295384 | 0.52367994 |
| 517 | 61 | 61 | 5.57 | 13.17 | 0.038962645 | 0.633625485 |
| 518 | 61 | 61 | 3.6 | 13.17 | 0.005151754 | 0.53583684 |
| 519 | 61 | 61 | 4.17 | 13.17 | 0.031192937 | 0.618203276 |
| 520 | 61 | 61 | 4.11 | 13.17 | 0.021853708 | 0.597030029 |
| 521 | 61 | 61 | 2.8 | 13.17 | 0.000769766 | 0.475861792 |
| 522 | 61 | 61 | 2.966666667 | 13.17 | 0.000126128 | 0.436862142 |
| 523 | 61 | 61 | 2.833333333 | 13.17 | 0.000580313 | 0.46691394 |
| 524 | 61 | 61 | 3.75 | 13.17 | 0.01187787 | 0.570766017 |
| 525 | 61 | 61 | 5.33 | 13.17 | 0.022326761 | 0.590489842 |
| 526 | 61 | 61 | 3.07 | 13.17 | 0.000840734 | 0.486615976 |
| 527 | 61 | 61 | 5.37 | 13.17 | 0.014952521 | 0.576588102 |
| 528 | 61 | 61 | 4.17 | 13.17 | 0.001544017 | 0.493221136 |
| 529 | 61 | 61 | 3.5 | 13.17 | 0.006074825 | 0.546145216 |
| 530 | 61 | 61 | 3.8 | 13.17 | 0.004085177 | 0.528374362 |
| 531 | 61 | 61 | 4.2 | 13.17 | 0.007450461 | 0.55721077 |
| 532 | 61 | 61 | 2.8 | 13.17 | 0.001351377 | 0.492374532 |
| 533 | 61 | 61 | 3.22 | 13.17 | 0.023439668 | 0.604673144 |
| 534 | 61 | 61 | 2.866666667 | 13.17 | 0.004534325 | 0.526116836 |
| 535 | 61 | 61 | 6.033333333 | 13.17 | 0.466633215 | 0.844276986 |
| 536 | 61 | 61 | 2.966666667 | 13.17 | 4.58E-05 | 0.409366707 |
| 537 | 61 | 61 | 5.57 | 13.17 | 0.014028806 | 0.579859218 |
| 538 | 61 | 61 | 3.166666667 | 13.17 | 0.010393342 | 0.565485665 |
| 539 | 61 | 61 | 3.333333333 | 13.17 | 0.009623011 | 0.563979532 |
| 540 | 61 | 61 | 4.4 | 13.17 | 0.04227266 | 0.632668371 |
| 541 | 61 | 61 | 5.43 | 13.17 | 0.052307451 | 0.635730147 |
| 542 | 61 | 61 | 3.26 | 13.17 | 0.000134275 | 0.428474134 |
| 543 | 61 | 61 | 4.63 | 13.17 | 0.001345873 | 0.492493371 |
| 544 | 61 | 61 | 3.6 | 13.17 | 0.000277566 | 0.445581491 |
| 545 | 61 | 61 | 3.266666667 | 13.17 | 0.011923914 | 0.574289488 |
| 546 | 61 | 61 | 4.17 | 13.17 | 0.012845905 | 0.577924534 |
| 547 | 61 | 61 | 3.22 | 13.17 | 0.004613202 | 0.530645205 |
| 548 | 61 | 61 | 2.833333333 | 13.17 | 0.007347138 | 0.551103499 |
| 549 | 61 | 61 | 3.07 | 13.17 | 0.000181874 | 0.433819904 |
| 550 | 61 | 61 | 3.77 | 13.17 | 0.000791424 | 0.483454984 |
| 551 | 61 | 61 | 3.17 | 13.17 | 0.00644793 | 0.551441354 |
| 552 | 61 | 61 | 3.95 | 13.17 | 0.027443483 | 0.609884353 |
| 553 | 61 | 61 | 2.96 | 13.17 | 0.000686091 | 0.465798023 |
| 554 | 61 | 61 | 3.333333333 | 13.17 | 0.016254881 | 0.578715275 |
| 555 | 61 | 61 | 3.68 | 13.17 | 0.054930609 | 0.649360914 |
| 556 | 61 | 61 | 3.42 | 13.17 | 0.006512199 | 0.547047225 |
| 557 | 61 | 61 | 4.27 | 13.17 | 0.005030051 | 0.535595951 |
| 558 | 61 | 61 | 4.133333333 | 13.17 | 0.039389396 | 0.62707558 |
| 559 | 61 | 61 | 3.91 | 13.17 | 0.015287521 | 0.577164854 |
| 560 | 61 | 61 | 4.24 | 13.17 | 0.013387901 | 0.569717528 |
| 561 | 61 | 61 | 4.05 | 13.17 | 0.024255499 | 0.599436101 |
| 562 | 61 | 61 | 3.29 | 13.17 | 0.005611476 | 0.543533715 |
| 563 | 61 | 61 | 3.68 | 13.17 | 0.019942908 | 0.585662985 |
| 564 | 61 | 61 | 3.65 | 13.17 | 0.012926429 | 0.572562741 |
| 565 | 61 | 61 | 2.67 | 13.17 | 4.04E-05 | 0.409257477 |
| 566 | 61 | 61 | 3.6 | 13.17 | 0.00289889 | 0.514633137 |
| 567 | 61 | 61 | 5.33 | 13.17 | 0.039782938 | 0.634596807 |
| 568 | 61 | 61 | 2.8 | 13.17 | 0.000144397 | 0.437501237 |
| 569 | 61 | 61 | 3.033333333 | 13.17 | 0.001567889 | 0.496661526 |
| 570 | 61 | 61 | 4.67 | 13.17 | 0.013559146 | 0.57526325 |
| 571 | 61 | 61 | 3.5 | 13.17 | 0.07436805 | 0.665514089 |
| 572 | 61 | 61 | 3.5 | 13.17 | 0.004273957 | 0.525637396 |
| 573 | 61 | 61 | 3.16 | 13.17 | 0.006409484 | 0.546057583 |
| 574 | 61 | 61 | 4.51 | 13.17 | 0.04693049 | 0.635232708 |
| 575 | 61 | 61 | 3.1 | 13.17 | 0.005251418 | 0.538533768 |
| 576 | 61 | 61 | 3.91 | 13.17 | 0.000647694 | 0.470106309 |
| 577 | 61 | 61 | 3.5 | 13.17 | 0.008641315 | 0.556401705 |
| 578 | 61 | 61 | 3.45 | 13.17 | 0.01284256 | 0.573679835 |
| 579 | 61 | 61 | 3.5 | 13.17 | 0.015681276 | 0.580661449 |
| 580 | 61 | 61 | 3.68 | 13.17 | 0.010852147 | 0.5700044 |
| 581 | 61 | 61 | 3.133333333 | 13.17 | 0.023786856 | 0.602583355 |
| 582 | 61 | 61 | 2.77 | 13.17 | 0.000416492 | 0.470846835 |
| 583 | 61 | 61 | 4.8 | 13.17 | 0.045054132 | 0.636987172 |
| 584 | 61 | 61 | 3.68 | 13.17 | 0.007333768 | 0.548833249 |
| 585 | 61 | 61 | 3.22 | 13.17 | 0.001862441 | 0.505066815 |
| 586 | 61 | 61 | 3.07 | 13.17 | 0.001109153 | 0.47983141 |
| 587 | 61 | 61 | 2.866666667 | 13.17 | 0.002469762 | 0.507685089 |
| 588 | 61 | 61 | 3.29 | 13.17 | 0.025067065 | 0.601177677 |
| 589 | 61 | 61 | 3.6 | 13.17 | 0.030401473 | 0.612022729 |
| 590 | 61 | 61 | 3.8 | 13.17 | 0.099530243 | 0.684330261 |
| 591 | 61 | 61 | 2.866666667 | 13.17 | 0.003557641 | 0.522817214 |
| 592 | 61 | 61 | 4.43 | 13.17 | 0.07902479 | 0.669680935 |
| 593 | 61 | 61 | 5.13 | 13.17 | 0.035734092 | 0.623670754 |
| 594 | 61 | 61 | 4.133333333 | 13.17 | 0.012441779 | 0.57263529 |
| 595 | 61 | 61 | 4.14 | 13.17 | 0.108648727 | 0.69346767 |
| 596 | 61 | 61 | 3.5 | 13.17 | 0.010496474 | 0.572051496 |
| 597 | 61 | 61 | 3.45 | 13.17 | 0.001389074 | 0.488166427 |
| 598 | 61 | 61 | 2.8 | 13.17 | 0.000236422 | 0.438987866 |
| 599 | 61 | 61 | 5.53 | 13.17 | 0.103948422 | 0.692349794 |
| 600 | 61 | 61 | 3.91 | 13.17 | 0.055823961 | 0.646628184 |
| 601 | 61 | 61 | 3.17 | 13.17 | 0.002932786 | 0.519327454 |
| 602 | 61 | 61 | 4 | 13.17 | 0.00870561 | 0.553137847 |
| 603 | 61 | 61 | 3.5 | 13.17 | 0.016509447 | 0.582817144 |
| 604 | 61 | 61 | 5.37 | 13.17 | 0.019828579 | 0.592097327 |
| 605 | 61 | 61 | 3.5 | 13.17 | 0.001916433 | 0.497826369 |
| 606 | 61 | 61 | 3.3 | 13.17 | 0.000802335 | 0.484819728 |
| 607 | 61 | 61 | 4.133333333 | 13.17 | 0.007078428 | 0.548334179 |
| 608 | 61 | 61 | 3.65 | 13.17 | 0.007490839 | 0.547581402 |
| 609 | 61 | 61 | 3.95 | 13.17 | 0.004176471 | 0.53201342 |
| 610 | 61 | 61 | 2.766666667 | 13.17 | 0.000704412 | 0.473562959 |
| 611 | 61 | 61 | 4.14 | 13.17 | 0.000391223 | 0.450476072 |
| 612 | 61 | 61 | 2.966666667 | 13.17 | 0.000324656 | 0.4541752 |
| 613 | 61 | 61 | 3.95 | 13.17 | 0.000950823 | 0.477866949 |
| 614 | 61 | 61 | 3.22 | 13.17 | 0.002790562 | 0.517186074 |
| 615 | 61 | 61 | 3.5 | 13.17 | 0.032781545 | 0.61829737 |
| 616 | 61 | 61 | 3.1 | 13.17 | 0.000594065 | 0.474234257 |
| 617 | 61 | 61 | 4.133333333 | 13.17 | 0.130340196 | 0.70345055 |
| 618 | 61 | 61 | 2.766666667 | 13.17 | 0.000340333 | 0.456160583 |
| 619 | 61 | 61 | 2.96 | 13.17 | 0.003240254 | 0.514099978 |
| 620 | 61 | 61 | 3.1 | 13.17 | 0.000966561 | 0.47701831 |
| 621 | 61 | 61 | 3.5 | 13.17 | 0.019714478 | 0.593964678 |
| 622 | 61 | 61 | 6.3 | 13.17 | 0.148270526 | 0.714495871 |
| 623 | 61 | 61 | 3.77 | 13.17 | 0.003738471 | 0.524611226 |
| 624 | 61 | 61 | 3.1 | 13.17 | 0.001588186 | 0.494743207 |
| 625 | 61 | 61 | 3.6 | 13.17 | 0.012950182 | 0.559695651 |
| 626 | 61 | 61 | 3.5 | 13.17 | 0.001564497 | 0.499664919 |
| 627 | 61 | 61 | 5.33 | 13.17 | 0.240482781 | 0.764056091 |
| 628 | 61 | 61 | 2.833333333 | 13.17 | 0.000978974 | 0.485980648 |
| 629 | 61 | 61 | 4.14 | 13.17 | 0.01236247 | 0.566820069 |
| 630 | 61 | 61 | 6.13 | 13.17 | 0.12726833 | 0.702799921 |
| 631 | 61 | 61 | 3.03 | 13.17 | 0.000748668 | 0.476051847 |
| 632 | 61 | 61 | 2.77 | 13.17 | 0.000283699 | 0.452976136 |
| 633 | 61 | 61 | 4.51 | 13.17 | 0.023330004 | 0.600912439 |
| 634 | 61 | 61 | 4.63 | 13.17 | 0.062428496 | 0.659207176 |
| 635 | 61 | 61 | 3.033333333 | 13.17 | 0.003087074 | 0.522272938 |
| 636 | 61 | 61 | 4.51 | 13.17 | 0.043173695 | 0.634528973 |
| 637 | 61 | 61 | 4.27 | 13.17 | 0.007448348 | 0.548131219 |
| 638 | 61 | 61 | 2.76 | 13.17 | 1.53E-06 | 0.346432513 |
| 639 | 61 | 61 | 3.42 | 13.17 | 0.001258893 | 0.490280941 |
| 640 | 61 | 61 | 2.96 | 13.17 | 5.85E-05 | 0.41514458 |
| 641 | 61 | 61 | 4.24 | 13.17 | 0.025072324 | 0.607922937 |
| 642 | 61 | 61 | 5.43 | 13.17 | 0.216588758 | 0.753469371 |
| 643 | 61 | 61 | 3.1 | 13.17 | 0.046220051 | 0.631731614 |
| 644 | 61 | 61 | 3.22 | 13.17 | 0.006863696 | 0.548417892 |
| 645 | 61 | 61 | 3.68 | 13.17 | 0.024777448 | 0.603414303 |
| 646 | 61 | 61 | 3.5 | 13.17 | 0.001420585 | 0.493319958 |
| 647 | 61 | 61 | 2.833333333 | 13.17 | 0.000650013 | 0.474166363 |
| 648 | 61 | 61 | 3.8 | 13.17 | 0.03447047 | 0.618802047 |
| 649 | 61 | 61 | 3.5 | 13.17 | 0.003833701 | 0.523426907 |
| 650 | 61 | 61 | 5.77 | 13.17 | 0.033717778 | 0.615847833 |
| 651 | 61 | 61 | 4.8 | 13.17 | 0.094230683 | 0.684960957 |
| 652 | 61 | 61 | 3.5 | 13.17 | 0.018138957 | 0.588662722 |
| 653 | 61 | 61 | 2.966666667 | 13.17 | 0.000530919 | 0.46490344 |
| 654 | 61 | 61 | 3.95 | 13.17 | 0.035953517 | 0.624778227 |
| 655 | 61 | 61 | 3.91 | 13.17 | 0.003391638 | 0.508075298 |
| 656 | 61 | 61 | 3.5 | 13.17 | 0.000415527 | 0.461780478 |
| 657 | 61 | 61 | 3.1 | 13.17 | 0.005642689 | 0.545744522 |
| 658 | 61 | 61 | 3.16 | 13.17 | 0.000145216 | 0.42802739 |
| 659 | 61 | 61 | 5.99 | 13.17 | 0.225802995 | 0.754996825 |
| 660 | 61 | 61 | 3.033333333 | 13.17 | 0.016172488 | 0.584469496 |
| 661 | 61 | 61 | 3.333333333 | 13.17 | 0.0043908 | 0.532858785 |
| 662 | 61 | 61 | 5.99 | 13.17 | 0.098240101 | 0.685564087 |
| 663 | 61 | 61 | 4.97 | 13.17 | 0.04952367 | 0.636115394 |
| 664 | 61 | 61 | 3.6 | 13.17 | 0.011787689 | 0.572732588 |
| 665 | 61 | 61 | 2.8 | 13.17 | 0.00142968 | 0.497435886 |
| 666 | 61 | 61 | 3.07 | 13.17 | 0.000659924 | 0.476276576 |
| 667 | 61 | 61 | 4.57 | 13.17 | 0.032297197 | 0.611103906 |
| 668 | 61 | 61 | 4.63 | 13.17 | 0.028339246 | 0.611227662 |
| 669 | 61 | 61 | 4.05 | 13.17 | 0.032233686 | 0.615674405 |
| 670 | 61 | 61 | 3.133333333 | 13.17 | 0.001924184 | 0.508484818 |
| 671 | 61 | 61 | 3.5 | 13.17 | 0.02179363 | 0.596457309 |
| 672 | 61 | 61 | 2.866666667 | 13.17 | 0.000470932 | 0.459941944 |
| 673 | 61 | 61 | 5.27 | 13.17 | 0.074606707 | 0.66735163 |
| 674 | 61 | 61 | 3.39 | 13.17 | 0.002085024 | 0.505456467 |
| 675 | 61 | 61 | 3.22 | 13.17 | 0.007199751 | 0.549121901 |
| 676 | 61 | 61 | 4.63 | 13.17 | 0.076801353 | 0.664151577 |
| 677 | 61 | 61 | 5.03 | 13.17 | 0.003745157 | 0.516316634 |
| 678 | 61 | 61 | 4.43 | 13.17 | 0.030451894 | 0.616419109 |
| 679 | 61 | 61 | 4.67 | 13.17 | 0.002811141 | 0.523979323 |
| 680 | 61 | 61 | 3.5 | 13.17 | 0.010259275 | 0.562564811 |
| 681 | 61 | 61 | 3.5 | 13.17 | 0.035938201 | 0.623873266 |
| 682 | 61 | 61 | 3.07 | 13.17 | 0.000397733 | 0.448029984 |
| 683 | 61 | 61 | 4.54 | 13.17 | 0.031759503 | 0.617387327 |
| 684 | 61 | 61 | 3.95 | 13.17 | 0.111187763 | 0.689459541 |
| 685 | 61 | 61 | 3.95 | 13.17 | 0.004946763 | 0.525494225 |
| 686 | 61 | 61 | 3.23 | 13.17 | 0.00091137 | 0.483807248 |
| 687 | 61 | 61 | 3.5 | 13.17 | 0.00785456 | 0.540752232 |
| 688 | 61 | 61 | 4.83 | 13.17 | 0.027486539 | 0.608150001 |
| 689 | 61 | 61 | 4.51 | 13.17 | 0.013984345 | 0.58285159 |
| 690 | 61 | 61 | 2.966666667 | 13.17 | 0.000198224 | 0.442414552 |
| 691 | 61 | 61 | 3.23 | 13.17 | 0.005190051 | 0.53647903 |
| 692 | 61 | 61 | 2.8 | 13.17 | 1.92E-05 | 0.399036511 |
| 693 | 61 | 61 | 3.52 | 13.17 | 0.009812641 | 0.56029045 |
| 694 | 61 | 61 | 2.966666667 | 13.17 | 0.004754792 | 0.536628506 |
| 695 | 61 | 61 | 3.52 | 13.17 | 0.01123599 | 0.574804394 |
| 696 | 61 | 61 | 3.6 | 13.17 | 0.040514387 | 0.631232209 |
| 697 | 61 | 61 | 4.7 | 13.17 | 0.046265232 | 0.639079133 |
| 698 | 61 | 61 | 3.6 | 13.17 | 0.017959816 | 0.594477595 |
| 699 | 61 | 61 | 4.73 | 13.17 | 0.025764018 | 0.599446653 |
| 700 | 61 | 61 | 3.1 | 13.17 | 0.001507981 | 0.501079039 |
| 701 | 61 | 61 | 5.43 | 13.17 | 0.158833916 | 0.723205152 |
| 702 | 61 | 61 | 3.033333333 | 13.17 | 0.000509888 | 0.464681332 |
| 703 | 61 | 61 | 3.22 | 13.17 | 0.013109694 | 0.570914194 |
| 704 | 61 | 61 | 2.833333333 | 13.17 | 4.12E-05 | 0.411574805 |
| 705 | 61 | 61 | 2.8 | 13.17 | 0.01069019 | 0.564117909 |
| 706 | 61 | 61 | 3.133333333 | 13.17 | 0.011245319 | 0.565673018 |
| 707 | 61 | 61 | 5.59 | 13.17 | 0.040122012 | 0.632096631 |
| 708 | 61 | 61 | 3.5 | 13.17 | 0.06067338 | 0.648444877 |
| 709 | 61 | 61 | 3.22 | 13.17 | 0.001785617 | 0.505532929 |
| 710 | 61 | 61 | 3.45 | 13.17 | 0.049096932 | 0.638022836 |
| 711 | 61 | 61 | 5.47 | 13.17 | 0.233518191 | 0.757939968 |
| 712 | 61 | 61 | 5.33 | 13.17 | 0.085957975 | 0.671868414 |
| 713 | 61 | 61 | 5.13 | 13.17 | 0.042666439 | 0.632721509 |
| 714 | 61 | 61 | 4.24 | 13.17 | 0.034634045 | 0.618826433 |
| 715 | 61 | 61 | 3.07 | 13.17 | 8.48E-05 | 0.416288324 |
| 716 | 61 | 61 | 4.7 | 13.17 | 0.038325199 | 0.627498695 |
| 717 | 61 | 61 | 3.95 | 13.17 | 0.007426899 | 0.549817195 |
| 718 | 61 | 61 | 5.43 | 13.17 | 0.089984195 | 0.676556907 |
| 719 | 61 | 61 | 5.37 | 13.17 | 0.095570643 | 0.682086564 |
| 720 | 61 | 61 | 3.6 | 13.17 | 0.062609099 | 0.655656877 |
| 721 | 61 | 61 | 3.43 | 13.17 | 0.01817402 | 0.592225338 |
| 722 | 61 | 61 | 4.27 | 13.17 | 0.002836353 | 0.50552389 |
| 723 | 61 | 61 | 2.9 | 13.17 | 0.000965268 | 0.486523298 |
| 724 | 61 | 61 | 3.19 | 13.17 | 0.00122746 | 0.478124706 |
| 725 | 61 | 61 | 2.8 | 13.17 | 0.000231994 | 0.447837314 |
| 726 | 61 | 61 | 4.54 | 13.17 | 0.014886041 | 0.578030106 |
| 727 | 61 | 61 | 3.17 | 13.17 | 2.55E-05 | 0.396784601 |
| 728 | 61 | 61 | 3.8 | 13.17 | 0.022674219 | 0.602495913 |
| 729 | 61 | 61 | 3.52 | 13.17 | 0.010618852 | 0.56430879 |
| 730 | 61 | 61 | 3.22 | 13.17 | 0.003711082 | 0.532689149 |
| 731 | 61 | 61 | 3.95 | 13.17 | 0.016079538 | 0.584482648 |
| 732 | 61 | 61 | 2.8 | 13.17 | 0.003006285 | 0.519454671 |
| 733 | 61 | 61 | 3.65 | 13.17 | 0.004646543 | 0.530607653 |
| 734 | 61 | 61 | 3.07 | 13.17 | 0.002760638 | 0.50601366 |
| 735 | 61 | 61 | 3.17 | 13.17 | 0.003919891 | 0.53002482 |
| 736 | 61 | 61 | 3.17 | 13.17 | 0.010193823 | 0.562341665 |
| 737 | 61 | 61 | 3.266666667 | 13.17 | 0.000966604 | 0.473497861 |
| 738 | 61 | 61 | 4.133333333 | 13.17 | 0.00809717 | 0.555246832 |
| 739 | 61 | 61 | 3.95 | 13.17 | 0.009389035 | 0.562551611 |
| 740 | 61 | 61 | 3.65 | 13.17 | 0.027226839 | 0.613149299 |
| 741 | 61 | 61 | 4.67 | 13.17 | 0.01386456 | 0.573407376 |
| 742 | 61 | 61 | 3.43 | 13.17 | 0.000585477 | 0.4762237 |
| 743 | 61 | 61 | 2.966666667 | 13.17 | 0.001915445 | 0.502629355 |
| 744 | 61 | 61 | 5.33 | 13.17 | 0.131694581 | 0.70802603 |
| 745 | 61 | 61 | 4.2 | 13.17 | 0.020108667 | 0.593365691 |
| 746 | 61 | 61 | 3.6 | 13.17 | 0.01041901 | 0.567955205 |
| 747 | 61 | 61 | 3.8 | 13.17 | 0.035788016 | 0.625870144 |
| 748 | 61 | 61 | 3.333333333 | 13.17 | 0.003356204 | 0.521884818 |
| 749 | 61 | 61 | 2.766666667 | 13.17 | 3.28E-06 | 0.362312367 |
| 750 | 61 | 61 | 3.6 | 13.17 | 0.040739294 | 0.630932305 |
| 751 | 61 | 61 | 4.27 | 13.17 | 0.029036459 | 0.612854097 |
| 752 | 61 | 61 | 3.5 | 13.17 | 0.001670383 | 0.495287862 |
| 753 | 61 | 61 | 3.133333333 | 13.17 | 0.000253525 | 0.448188169 |
| 754 | 61 | 61 | 4.4 | 13.17 | 0.013292467 | 0.573352647 |
| 755 | 61 | 61 | 4.4 | 13.17 | 0.009893572 | 0.558524304 |
| 756 | 61 | 61 | 4.54 | 13.17 | 0.05452514 | 0.645344259 |
| 757 | 61 | 61 | 2.966666667 | 13.17 | 3.18E-05 | 0.402243666 |
| 758 | 61 | 61 | 3.1 | 13.17 | 0.005014014 | 0.533370068 |
| 759 | 61 | 61 | 4.54 | 13.17 | 0.148310468 | 0.718711071 |
| 760 | 61 | 61 | 3.5 | 13.17 | 0.005423643 | 0.539614634 |
| 761 | 61 | 61 | 2.8 | 13.17 | 0.003271787 | 0.523814171 |
| 762 | 61 | 61 | 3.42 | 13.17 | 0.011849804 | 0.568910932 |
| 763 | 61 | 61 | 2.866666667 | 13.17 | 0.00018587 | 0.441440853 |
| 764 | 61 | 61 | 4.11 | 13.17 | 0.01550659 | 0.579331199 |
| 765 | 61 | 61 | 3.52 | 13.17 | 0.010614345 | 0.568320585 |
| 766 | 61 | 61 | 4.17 | 13.17 | 0.023650825 | 0.605182009 |
| 767 | 61 | 61 | 3.45 | 13.17 | 0.002266549 | 0.506540396 |
| 768 | 61 | 61 | 4.17 | 13.17 | 0.006381878 | 0.554270252 |
| 769 | 61 | 61 | 3.133333333 | 13.17 | 0.00514029 | 0.533888784 |
| 770 | 61 | 61 | 3.033333333 | 13.17 | 0.000139674 | 0.429664837 |
| 771 | 61 | 61 | 2.966666667 | 13.17 | 0.007063067 | 0.549238648 |
| 772 | 61 | 61 | 4 | 13.17 | 0.022251868 | 0.598764312 |
| 773 | 61 | 61 | 3.6 | 13.17 | 0.010746457 | 0.571781779 |
| 774 | 61 | 61 | 3.266666667 | 13.17 | 0.015578925 | 0.579391559 |
| 775 | 61 | 61 | 4.4 | 13.17 | 0.009063107 | 0.557576104 |
| 776 | 61 | 61 | 4.83 | 13.17 | 0.012474475 | 0.574322738 |
| 777 | 61 | 61 | 4.33 | 13.17 | 0.015664812 | 0.583294848 |
| 778 | 61 | 61 | 2.9 | 13.17 | 0.000394394 | 0.463398931 |
| 779 | 61 | 61 | 2.66 | 13.17 | 1.65E-05 | 0.390742484 |
| 780 | 61 | 61 | 3.43 | 13.17 | 0.011239823 | 0.565687687 |
| 781 | 61 | 61 | 3.6 | 13.17 | 0.002466257 | 0.508516008 |
| 782 | 61 | 61 | 3.91 | 13.17 | 0.003912612 | 0.535356241 |
| 783 | 61 | 61 | 3.8 | 13.17 | 0.015130224 | 0.57630965 |
| 784 | 61 | 61 | 4.73 | 13.17 | 0.134450053 | 0.70858825 |
| 785 | 61 | 61 | 4.73 | 13.17 | 0.037292789 | 0.620477452 |
| 786 | 61 | 61 | 3.95 | 13.17 | 0.004319673 | 0.519946593 |
| 787 | 61 | 61 | 4.4 | 13.17 | 0.030827238 | 0.616013008 |
| 788 | 61 | 61 | 2.9 | 13.17 | 0.006890611 | 0.546360165 |
| 789 | 61 | 61 | 3.17 | 13.17 | 0.005555698 | 0.535752536 |
| 790 | 61 | 61 | 3.5 | 13.17 | 0.020210951 | 0.593306925 |
| 791 | 61 | 61 | 2.833333333 | 13.17 | 0.001946993 | 0.507375346 |
| 792 | 61 | 61 | 4 | 13.17 | 0.02710479 | 0.609657472 |
| 793 | 61 | 61 | 5.47 | 13.17 | 0.141924579 | 0.713204469 |
| 794 | 61 | 61 | 3.5 | 13.17 | 0.001201158 | 0.497199393 |
| 795 | 61 | 61 | 3.77 | 13.17 | 0.023491311 | 0.603050392 |
| 796 | 61 | 61 | 5.37 | 13.17 | 0.048725938 | 0.638984094 |
| 797 | 61 | 61 | 2.766666667 | 13.17 | 0.00283794 | 0.517948431 |
| 798 | 61 | 61 | 5.833333333 | 13.17 | 0.00998383 | 0.55969768 |
| 799 | 61 | 61 | 3.5 | 13.17 | 0.081896904 | 0.671813194 |
| 800 | 61 | 61 | 5.13 | 13.17 | 0.182616193 | 0.736369353 |
| 801 | 61 | 61 | 3.3 | 13.17 | 0.017137247 | 0.583407789 |
| 802 | 61 | 61 | 3.95 | 13.17 | 0.041386921 | 0.63194963 |
| 803 | 61 | 61 | 4.2 | 13.17 | 0.020556697 | 0.595933548 |
| 804 | 61 | 61 | 3.07 | 13.17 | 0.000374753 | 0.458033113 |
| 805 | 61 | 61 | 2.93 | 13.17 | 0.010332327 | 0.564134755 |
| 806 | 61 | 61 | 3.133333333 | 13.17 | 0.001417817 | 0.488675808 |
| 807 | 61 | 61 | 3.5 | 13.17 | 0.005842303 | 0.548306175 |
| 808 | 61 | 61 | 3.65 | 13.17 | 0.000241471 | 0.447857513 |
| 809 | 61 | 61 | 4.97 | 13.17 | 0.003401758 | 0.522913745 |
| 810 | 61 | 61 | 4.37 | 13.17 | 0.04238739 | 0.633333178 |
| 811 | 61 | 61 | 5.37 | 13.17 | 0.065588877 | 0.660560029 |
| 812 | 61 | 61 | 3.3 | 13.17 | 0.004812966 | 0.535650303 |
| 813 | 61 | 61 | 4.7 | 13.17 | 0.03588136 | 0.626958779 |
| 814 | 61 | 61 | 3.6 | 13.17 | 0.010553537 | 0.570151468 |
| 815 | 61 | 61 | 4.54 | 13.17 | 0.069869337 | 0.655790549 |
| 816 | 61 | 61 | 4.17 | 13.17 | 0.000319439 | 0.454057327 |
| 817 | 61 | 61 | 5.47 | 13.17 | 0.039730165 | 0.628751802 |
| 818 | 61 | 61 | 4.33 | 13.17 | 0.089973237 | 0.681879202 |
| 819 | 61 | 61 | 3.23 | 13.17 | 0.002977396 | 0.519328536 |
| 820 | 61 | 61 | 5.4 | 13.17 | 0.06905626 | 0.662671218 |
| 821 | 61 | 61 | 3.29 | 13.17 | 0.013466219 | 0.573492414 |
| 822 | 61 | 61 | 3.49 | 13.17 | 0.030243585 | 0.614265471 |
| 823 | 61 | 61 | 5.77 | 13.17 | 0.079738347 | 0.66843397 |
| 824 | 61 | 61 | 4.37 | 13.17 | 0.030322997 | 0.609139014 |
| 825 | 61 | 61 | 6.28 | 13.17 | 0.126472473 | 0.703340527 |
| 826 | 61 | 61 | 5.53 | 13.17 | 0.131396359 | 0.710241757 |
| 827 | 61 | 61 | 3.91 | 13.17 | 0.015586788 | 0.579066605 |
| 828 | 61 | 61 | 3.07 | 13.17 | 8.43E-05 | 0.419835044 |
| 829 | 61 | 61 | 5.27 | 13.17 | 0.079219099 | 0.669266747 |
| 830 | 61 | 61 | 3.6 | 13.17 | 0.017632661 | 0.586593557 |
| 831 | 61 | 61 | 3.95 | 13.17 | 0.056903754 | 0.650573675 |
| 832 | 61 | 61 | 3.65 | 13.17 | 0.024421867 | 0.601163934 |
| 833 | 61 | 61 | 2.833333333 | 13.17 | 0.001167244 | 0.495045512 |
| 834 | 61 | 61 | 4.73 | 13.17 | 0.077282294 | 0.669596286 |
| 835 | 61 | 61 | 3.45 | 13.17 | 0.001282717 | 0.476881239 |
| 836 | 61 | 61 | 4.4 | 13.17 | 0.011295243 | 0.564545388 |
| 837 | 61 | 61 | 5.77 | 13.17 | 0.351438416 | 0.802945216 |
| 838 | 61 | 61 | 4.14 | 13.17 | 0.006791312 | 0.55116273 |
| 839 | 61 | 61 | 2.9 | 13.17 | 0.000532098 | 0.466251104 |
| 840 | 61 | 61 | 3.1 | 13.17 | 0.000163227 | 0.435306648 |
| 841 | 61 | 61 | 2.833333333 | 13.17 | 0.001061373 | 0.486523038 |
| 842 | 61 | 61 | 4.27 | 13.17 | 0.01983233 | 0.595588751 |
| 843 | 61 | 61 | 6.03 | 13.17 | 0.114766138 | 0.69219056 |
| 844 | 61 | 61 | 2.833333333 | 13.17 | 8.86E-05 | 0.414864342 |
| 845 | 61 | 61 | 3.16 | 13.17 | 0.005164286 | 0.539741569 |
| 846 | 61 | 61 | 3.42 | 13.17 | 0.004066559 | 0.529212669 |
| 847 | 61 | 61 | 2.66 | 13.17 | 1.53E-05 | 0.395070572 |
| 848 | 61 | 61 | 3.91 | 13.17 | 0.012860532 | 0.57430739 |
| 849 | 61 | 61 | 4.05 | 13.17 | 0.013990561 | 0.575378954 |
| 850 | 61 | 61 | 4.57 | 13.17 | 0.029713151 | 0.614138245 |
| 851 | 61 | 61 | 3.6 | 13.17 | 0.007038581 | 0.545903559 |
| 852 | 61 | 61 | 3.033333333 | 13.17 | 0.002712797 | 0.517930842 |
| 853 | 61 | 61 | 3.1 | 13.17 | 0.001686622 | 0.502004243 |
| 854 | 61 | 61 | 3.5 | 13.17 | 0.02201263 | 0.593945136 |
| 855 | 61 | 61 | 3.5 | 13.17 | 0.00208592 | 0.503428052 |
| 856 | 61 | 61 | 2.8 | 13.17 | 0.001449345 | 0.484848492 |
| 857 | 61 | 61 | 4.41 | 13.17 | 0.081138643 | 0.672415478 |
| 858 | 61 | 61 | 2.8 | 13.17 | 0.000124437 | 0.426753346 |
| 859 | 61 | 61 | 4.133333333 | 13.17 | 0.000813545 | 0.47644152 |
| 860 | 61 | 61 | 3.8 | 13.17 | 0.006432847 | 0.549411828 |
| 861 | 61 | 61 | 3.5 | 13.17 | 0.002488273 | 0.509466327 |
| 862 | 61 | 61 | 4.27 | 13.17 | 0.028236622 | 0.608601678 |
| 863 | 61 | 61 | 3.07 | 13.17 | 0.000130816 | 0.436910101 |
| 864 | 61 | 61 | 4.17 | 13.17 | 0.041188877 | 0.62266641 |
| 865 | 61 | 61 | 3.17 | 13.17 | 0.002114196 | 0.505297035 |
| 866 | 61 | 61 | 3.03 | 13.17 | 7.28E-05 | 0.415652336 |
| 867 | 61 | 61 | 3.17 | 13.17 | 0.000272785 | 0.45704437 |
| 868 | 61 | 61 | 3.65 | 13.17 | 0.050612468 | 0.643828817 |
| 869 | 61 | 61 | 3.166666667 | 13.17 | 0.001481716 | 0.486840883 |
| 870 | 61 | 61 | 4.27 | 13.17 | 0.028955082 | 0.615794461 |
| 871 | 61 | 61 | 3.5 | 13.17 | 0.001648169 | 0.496864749 |
| 872 | 61 | 61 | 3.22 | 13.17 | 0.010406632 | 0.567766167 |
| 873 | 61 | 61 | 3.266666667 | 13.17 | 0.014096138 | 0.575024781 |
| 874 | 61 | 61 | 3.49 | 13.17 | 0.002311775 | 0.509114017 |
| 875 | 61 | 61 | 3.39 | 13.17 | 0.001070758 | 0.486235564 |
| 876 | 61 | 61 | 3.22 | 13.17 | 0.001057901 | 0.49493115 |
| 877 | 61 | 61 | 3.133333333 | 13.17 | 0.000112344 | 0.422569154 |
| 878 | 61 | 61 | 3.8 | 13.17 | 0.000362261 | 0.458475036 |
| 879 | 61 | 61 | 4.41 | 13.17 | 0.101806758 | 0.686850786 |
| 880 | 61 | 61 | 4.37 | 13.17 | 0.015176339 | 0.582629883 |
| 881 | 61 | 61 | 3.133333333 | 13.17 | 0.000761247 | 0.477626361 |
| 882 | 61 | 61 | 2.966666667 | 13.17 | 0.002797346 | 0.520958913 |
| 883 | 61 | 61 | 3.5 | 13.17 | 0.033760534 | 0.6141221 |
| 884 | 61 | 61 | 4.05 | 13.17 | 0.018665096 | 0.592534844 |
| 885 | 61 | 61 | 3.95 | 13.17 | 0.000881363 | 0.46721046 |
| 886 | 61 | 61 | 3.95 | 13.17 | 0.009233389 | 0.560572497 |
| 887 | 61 | 61 | 3.77 | 13.17 | 0.018809396 | 0.594418152 |
| 888 | 61 | 61 | 3.65 | 13.17 | 0.002004918 | 0.50500791 |
| 889 | 61 | 61 | 2.866666667 | 13.17 | 0.001398088 | 0.493025683 |
| 890 | 61 | 61 | 3.45 | 13.17 | 0.006298633 | 0.546736429 |
| 891 | 61 | 61 | 3.26 | 13.17 | 0.006352325 | 0.547987043 |
| 892 | 61 | 61 | 4.11 | 13.17 | 0.08053196 | 0.669931383 |
| 893 | 61 | 61 | 2.66 | 13.17 | 8.36E-05 | 0.424675445 |
| 894 | 61 | 61 | 4.57 | 13.17 | 0.113898992 | 0.699690385 |
| 895 | 61 | 61 | 2.96 | 13.17 | 0.002350709 | 0.506463051 |
| 896 | 61 | 61 | 3.6 | 13.17 | 0.02177771 | 0.59483976 |
| 897 | 61 | 61 | 3.75 | 13.17 | 0.011314743 | 0.571657 |
| 898 | 61 | 61 | 3.5 | 13.17 | 0.019292518 | 0.594321594 |
| 899 | 61 | 61 | 3.07 | 13.17 | 0.000454977 | 0.462460133 |
| 900 | 61 | 61 | 5.4 | 13.17 | 0.020909312 | 0.591334835 |
| 901 | 61 | 61 | 3.5 | 13.17 | 0.016513242 | 0.583384433 |
| 902 | 61 | 61 | 4.43 | 13.17 | 0.00735461 | 0.549062331 |
| 903 | 61 | 61 | 5.1 | 13.17 | 0.03810129 | 0.624236933 |
| 904 | 61 | 61 | 3.07 | 13.17 | 0.000675202 | 0.46880126 |
| 905 | 61 | 61 | 3.6 | 13.17 | 0.008316249 | 0.55728965 |
| 906 | 61 | 61 | 4.433333333 | 13.17 | 0.152304085 | 0.720821152 |
| 907 | 61 | 61 | 3.266666667 | 13.17 | 0.056572676 | 0.652052398 |
| 908 | 61 | 61 | 2.766666667 | 13.17 | 0.001059499 | 0.48757108 |
| 909 | 61 | 61 | 4.17 | 13.17 | 0.021568896 | 0.601827474 |
| 910 | 61 | 61 | 4.27 | 13.17 | 0.020976429 | 0.598869684 |
| 911 | 61 | 61 | 4.37 | 13.17 | 0.032507729 | 0.617513245 |
| 912 | 61 | 61 | 3.65 | 13.17 | 0.059163083 | 0.646156131 |
| 913 | 61 | 61 | 3.1 | 13.17 | 0.0064986 | 0.547913428 |
| 914 | 61 | 61 | 2.966666667 | 13.17 | 0.000330335 | 0.449674956 |
| 915 | 61 | 61 | 3.22 | 13.17 | 0.00150687 | 0.499577718 |
| 916 | 61 | 61 | 3.266666667 | 13.17 | 0.011909199 | 0.567216736 |
| 917 | 61 | 61 | 3.03 | 13.17 | 0.000652285 | 0.473026987 |
| 918 | 61 | 61 | 5.47 | 13.17 | 0.077912727 | 0.67202433 |
| 919 | 61 | 61 | 4.17 | 13.17 | 0.010518278 | 0.567238661 |
| 920 | 61 | 61 | 3.17 | 13.17 | 0.000712222 | 0.472722705 |
| 921 | 61 | 61 | 3.1 | 13.17 | 0.001831618 | 0.503076369 |
| 922 | 61 | 61 | 3.29 | 13.17 | 0.001947686 | 0.508552048 |
| 923 | 61 | 61 | 3.77 | 13.17 | 0.006409818 | 0.544058518 |
| 924 | 61 | 61 | 4.11 | 13.17 | 0.004386387 | 0.528976059 |
| 925 | 61 | 61 | 3.29 | 13.17 | 0.001449026 | 0.497439838 |
| 926 | 61 | 61 | 5.1 | 13.17 | 0.026637707 | 0.612330821 |
| 927 | 61 | 61 | 4.7 | 13.17 | 0.098066316 | 0.688302076 |
| 928 | 61 | 61 | 2.9 | 13.17 | 0.000829895 | 0.485939663 |
| 929 | 61 | 61 | 3.8 | 13.17 | 0.001039921 | 0.487106432 |
| 930 | 61 | 61 | 3.68 | 13.17 | 0.010712008 | 0.568630003 |
| 931 | 61 | 61 | 4.33 | 13.17 | 0.002408968 | 0.50828663 |
| 932 | 61 | 61 | 7.2 | 13.17 | 0.321807734 | 0.791767837 |
| 933 | 61 | 61 | 3.19 | 13.17 | 0.005721938 | 0.541279997 |
| 934 | 61 | 61 | 4.54 | 13.17 | 0.065702493 | 0.656701066 |
| 935 | 61 | 61 | 3.5 | 13.17 | 0.008617893 | 0.559024672 |
| 936 | 61 | 61 | 3.29 | 13.17 | 0.000940329 | 0.480408954 |
| 937 | 61 | 61 | 3.6 | 13.17 | 0.00568427 | 0.539485647 |
| 938 | 61 | 61 | 3.42 | 13.17 | 0.000647513 | 0.473688114 |
| 939 | 61 | 61 | 4.41 | 13.17 | 0.081715757 | 0.670255294 |
| 940 | 61 | 61 | 4.37 | 13.17 | 0.001325959 | 0.487369758 |
| 941 | 61 | 61 | 3.39 | 13.17 | 0.000163301 | 0.431891068 |
| 942 | 61 | 61 | 4.27 | 13.17 | 0.026314032 | 0.608867757 |
| 943 | 61 | 61 | 4.67 | 13.17 | 0.074275084 | 0.667420928 |
| 944 | 61 | 61 | 4.133333333 | 13.17 | 0.007067978 | 0.553152238 |
| 945 | 61 | 61 | 4.33 | 13.17 | 0.018031252 | 0.586052176 |
| 946 | 61 | 61 | 2.93 | 13.17 | 9.48E-05 | 0.422145504 |
| 947 | 61 | 61 | 4.51 | 13.17 | 0.068733505 | 0.664465178 |
| 948 | 61 | 61 | 4.8 | 13.17 | 0.094379861 | 0.683491833 |
| 949 | 61 | 61 | 2.73 | 13.17 | 2.15E-05 | 0.398230157 |
| 950 | 61 | 61 | 3.29 | 13.17 | 0.016267988 | 0.583517789 |
| 951 | 61 | 61 | 3.19 | 13.17 | 0.002991974 | 0.5237239 |
| 952 | 61 | 61 | 3.75 | 13.17 | 0.008599297 | 0.554362132 |
| 953 | 61 | 61 | 4.51 | 13.17 | 0.006345925 | 0.543166985 |
| 954 | 61 | 61 | 5.27 | 13.17 | 0.040608464 | 0.631925275 |
| 955 | 61 | 61 | 2.8 | 13.17 | 0.009851936 | 0.560433776 |
| 956 | 61 | 61 | 2.8 | 13.17 | 0.031175896 | 0.610909041 |
| 957 | 61 | 61 | 5.53 | 13.17 | 0.042729976 | 0.632558215 |
| 958 | 61 | 61 | 3.42 | 13.17 | 0.017675741 | 0.590503121 |
| 959 | 61 | 61 | 3.17 | 13.17 | 0.011041226 | 0.571127416 |
| 960 | 61 | 61 | 3.77 | 13.17 | 0.005901766 | 0.546953628 |
| 961 | 61 | 61 | 3.22 | 13.17 | 0.001606226 | 0.494148053 |
| 962 | 61 | 61 | 4.73 | 13.17 | 0.038450005 | 0.627043892 |
| 963 | 61 | 61 | 4.17 | 13.17 | 0.030118169 | 0.615361742 |
| 964 | 61 | 61 | 2.73 | 13.17 | 0.00014767 | 0.435522275 |
| 965 | 61 | 61 | 3.22 | 13.17 | 0.004134443 | 0.531973976 |
| 966 | 61 | 61 | 2.8 | 13.17 | 0.000762425 | 0.478062076 |
| 967 | 61 | 61 | 2.8 | 13.17 | 0.000301814 | 0.454987411 |
| 968 | 61 | 61 | 3.033333333 | 13.17 | 0.001205607 | 0.480819798 |
| 969 | 61 | 61 | 4.27 | 13.17 | 0.002141678 | 0.502066672 |
| 970 | 61 | 61 | 4.33 | 13.17 | 0.012515019 | 0.57196126 |
| 971 | 61 | 61 | 3.5 | 13.17 | 0.009559743 | 0.564401921 |
| 972 | 61 | 61 | 4.8 | 13.17 | 0.023957087 | 0.604600156 |
| 973 | 61 | 61 | 2.9 | 13.17 | 0.006223162 | 0.545838683 |
| 974 | 61 | 61 | 3.5 | 13.17 | 0.0088101 | 0.547518107 |
| 975 | 61 | 61 | 7.233333333 | 13.17 | 0.024384364 | 0.605217633 |
| 976 | 61 | 61 | 4.05 | 13.17 | 0.053085899 | 0.643061281 |
| 977 | 61 | 61 | 4.33 | 13.17 | 0.125453329 | 0.707030329 |
| 978 | 61 | 61 | 3.29 | 13.17 | 0.019268546 | 0.587400741 |
| 979 | 61 | 61 | 4.14 | 13.17 | 0.000735661 | 0.473685294 |
| 980 | 61 | 61 | 4.63 | 13.17 | 0.008701068 | 0.561243447 |
| 981 | 61 | 61 | 3.333333333 | 13.17 | 0.006408601 | 0.545907069 |
| 982 | 61 | 61 | 4.83 | 13.17 | 0.154316805 | 0.722521279 |
| 983 | 61 | 61 | 6.81 | 13.17 | 0.105788621 | 0.694378737 |
| 984 | 61 | 61 | 2.9 | 13.17 | 0.00028586 | 0.451475669 |
| 985 | 61 | 61 | 4.83 | 13.17 | 0.025524723 | 0.608615761 |
| 986 | 61 | 61 | 3.6 | 13.17 | 0.000567741 | 0.470610498 |
| 987 | 61 | 61 | 5.43 | 13.17 | 0.161316517 | 0.724387537 |
| 988 | 61 | 61 | 4.17 | 13.17 | 0.032388811 | 0.616036152 |
| 989 | 61 | 61 | 3.266666667 | 13.17 | 4.72E-05 | 0.408680951 |
| 990 | 61 | 61 | 5.47 | 13.17 | 0.04272259 | 0.632807341 |
| 991 | 61 | 61 | 2.8 | 13.17 | 0.002911664 | 0.514543787 |
| 992 | 61 | 61 | 5.13 | 13.17 | 0.020292868 | 0.590483519 |
| 993 | 61 | 61 | 3.52 | 13.17 | 0.001701836 | 0.501317591 |
| 994 | 61 | 61 | 3.17 | 13.17 | 0.000162303 | 0.427293731 |
| 995 | 61 | 61 | 4.27 | 13.17 | 0.017366796 | 0.586165384 |
| 996 | 61 | 61 | 5.27 | 13.17 | 0.048173039 | 0.633859993 |
| 997 | 61 | 61 | 3.5 | 13.17 | 0.008463832 | 0.551022243 |
| 998 | 61 | 61 | 4.14 | 13.17 | 0.035494835 | 0.626585214 |
| 999 | 61 | 61 | 3.6 | 13.17 | 0.008302135 | 0.560752469 |
| 1000 | 61 | 61 | 3.17 | 13.17 | 0.000618025 | 0.474576139 |
