# Supplementary figures and images for: Identification of NOTCH4 mutation as a response biomarker for immune checkpoint inhibitor therapy
Source: BMC Med. 2021 Jul 21;19:154. doi: 10.1186/s12916-021-02031-3 (PMC8293505; doi:10.1186/s12916-021-02031-3)

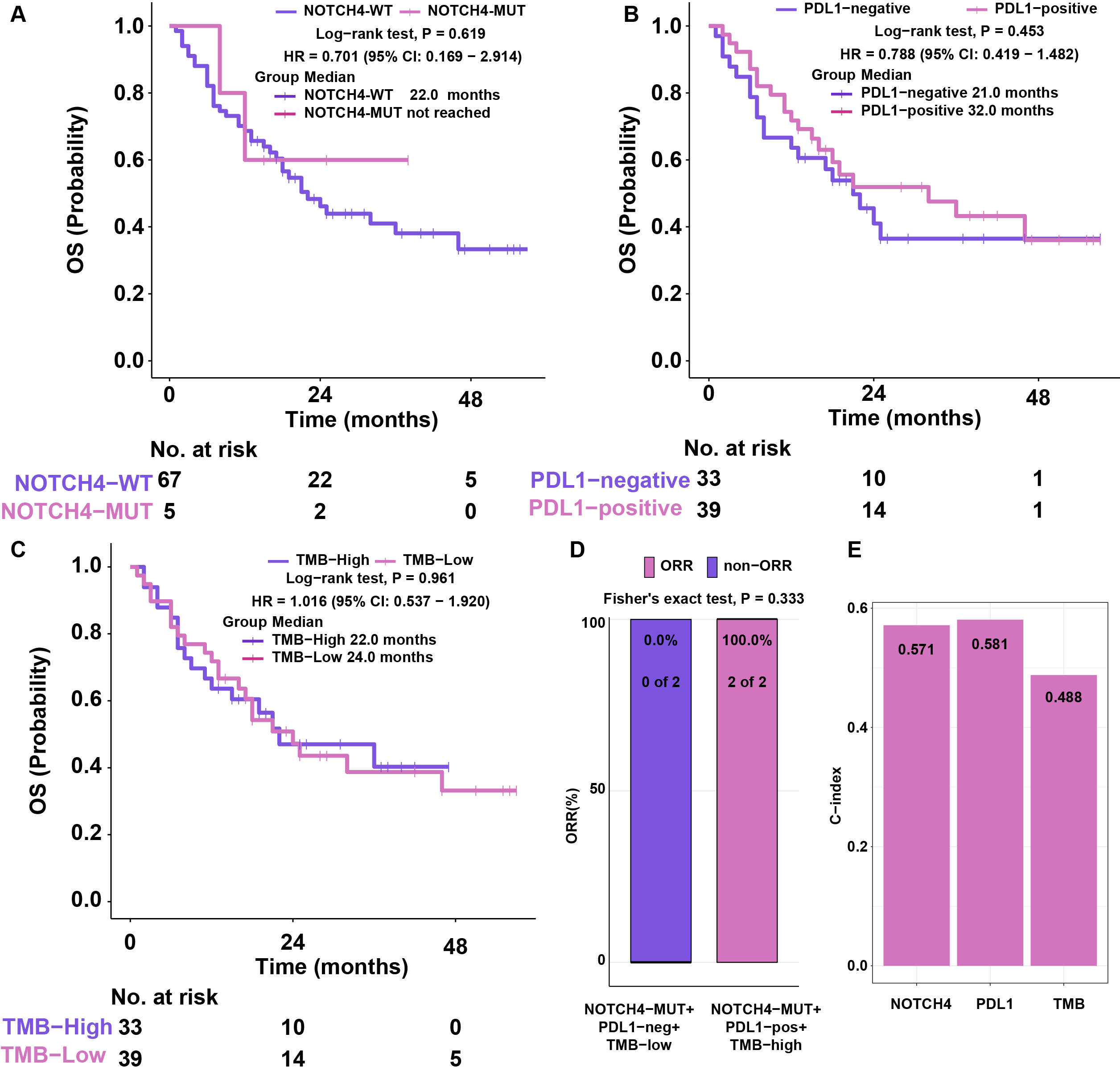

Supplement: Supplementary file 2 — Additional file 2. Figure S1. Relationships among NOTCH4 status, PD-L1 status and TMB. (A) Predictive value of NOTCH4 mutation for the overall survival (OS) of NSCLC patients in the discovery cohort. (B) Predictive value of PD-L1 for the OS of NSCLC patients in the discovery cohort. (C) Predictive value of TMB for the OS of NSCLC patients in the discovery cohort. (D) Histogram showing the proportions of patients who achieved an objective response rate (ORR) among NOTCH4 MUT/PD-L1-negative/TMB-low and NOTCH4 MUT/PD-L1-positive/TMB-high patients. (E) Histogram showing the C-index of NOTCH4 status, PD-L1 status and TMB. [file 12916_2021_2031_MOESM2_ESM.tif]

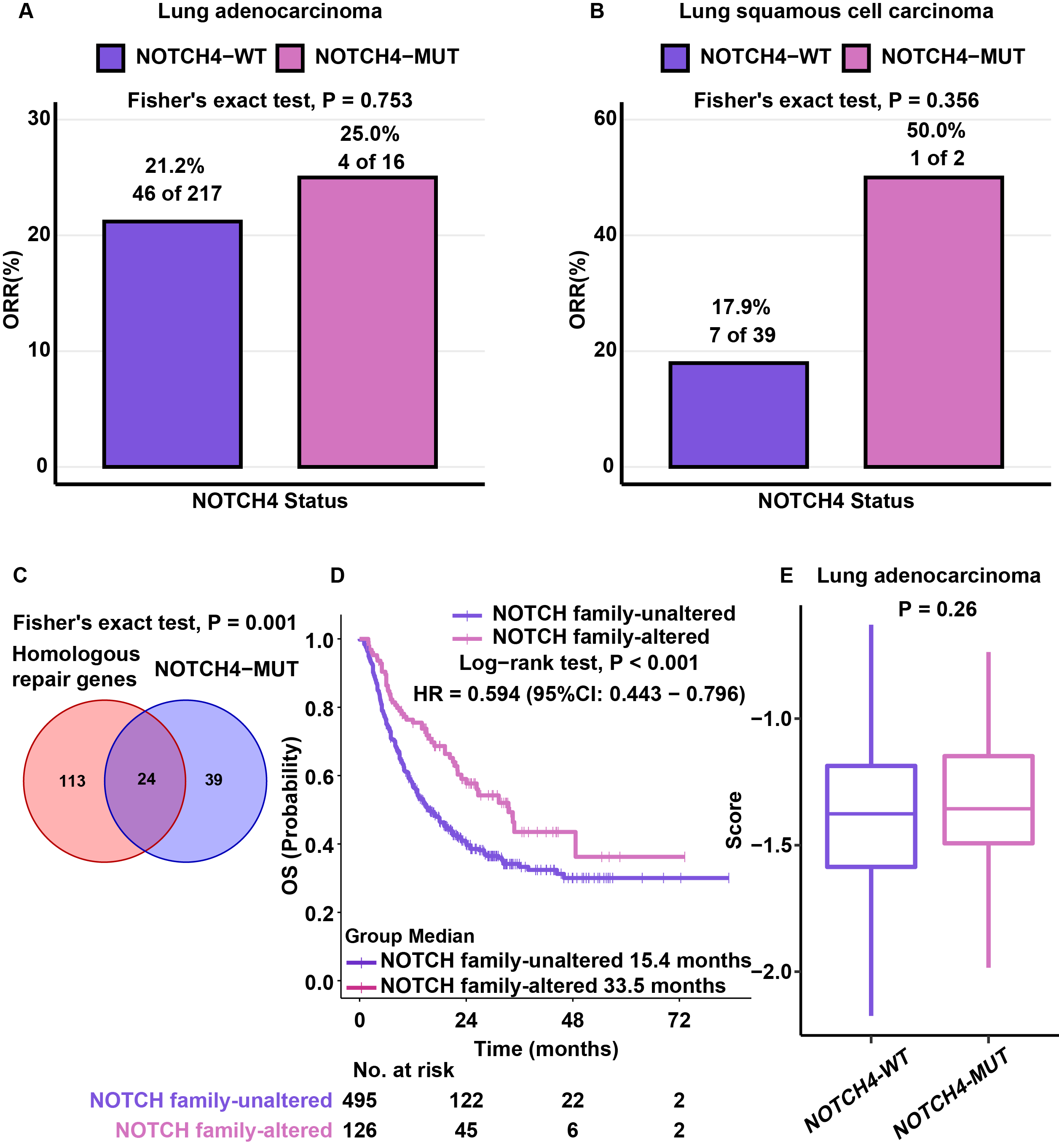

Supplement: Supplementary file 3 — Additional file 3. Figure S2. Relationships between NOTCH4 status and objective response rate in lung adenocarcinoma and lung squamous cell carcinoma. (A) Histogram showing the proportions of patients who achieved an objective response rate (ORR) among NOTCH4-WT and NOTCH4-MUT patients with lung adenocarcinoma. (B) Histogram showing the proportions of patients who achieved an ORR among NOTCH4-WT and NOTCH4-MUT patients with lung squamous cell carcinoma. (C) Venn diagram showing the overlap between homologous repair gene mutations and NOTCH4 mutation in the discovery cohort. (D) Predictive value of NOTCH4 family mutation for overall survival (OS) in the discovery cohort. (E) Comparison of the NOTCH pathway score between NOTCH4-WT and NOTCH4-MUT in patients with lung adenocarcinoma in the TCGA dataset. [file 12916_2021_2031_MOESM3_ESM.tif]

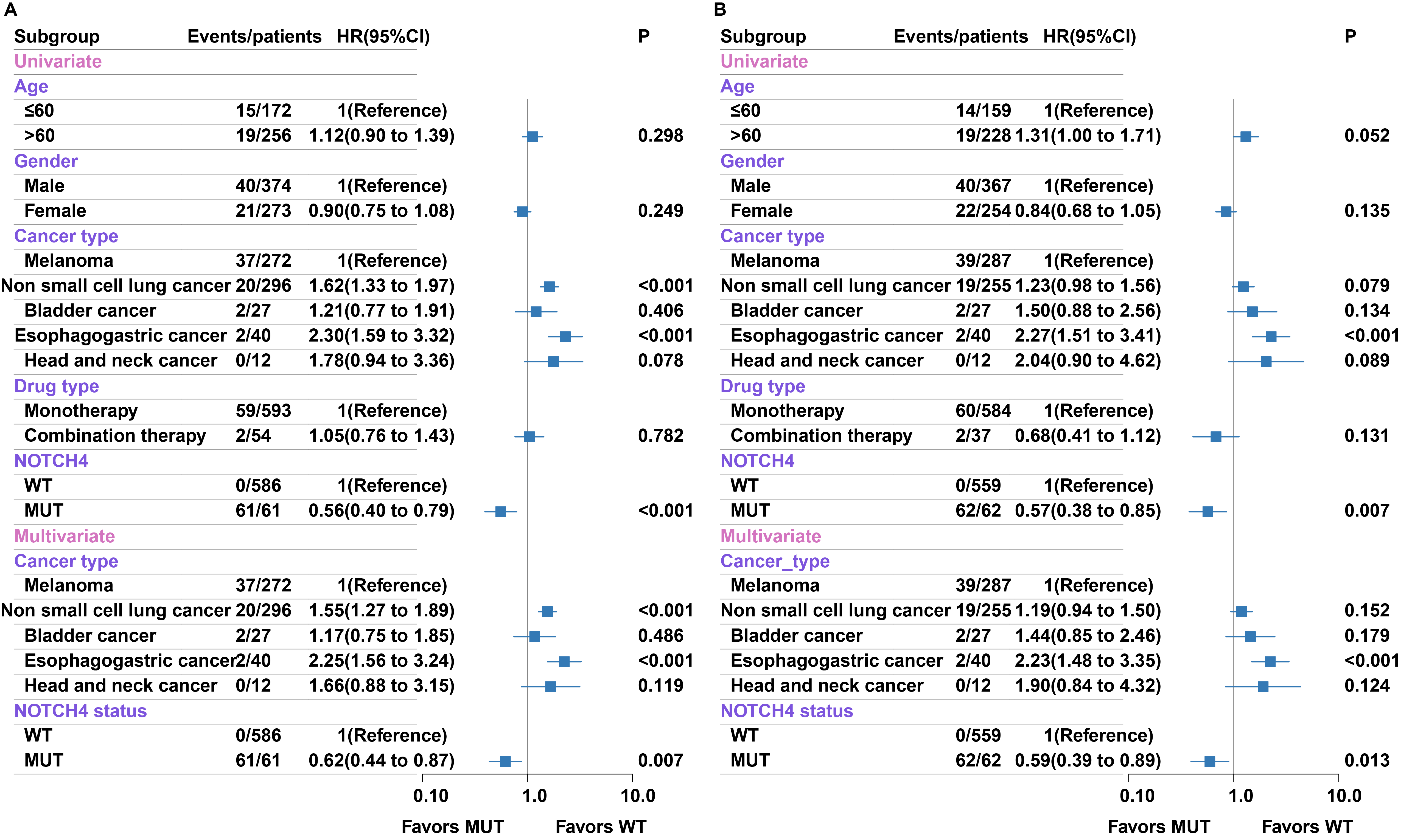

Supplement: Supplementary file 5 — Additional file 5. Figure S3. Relationships between NOTCH4 status and other characteristics. (A) Univariate and multivariate analyses of progression-free survival (PFS) according to NOTCH4 status. (B) Univariate and multivariate analyses of overall survival (OS) according to NOTCH4 status. [file 12916_2021_2031_MOESM5_ESM.tif]

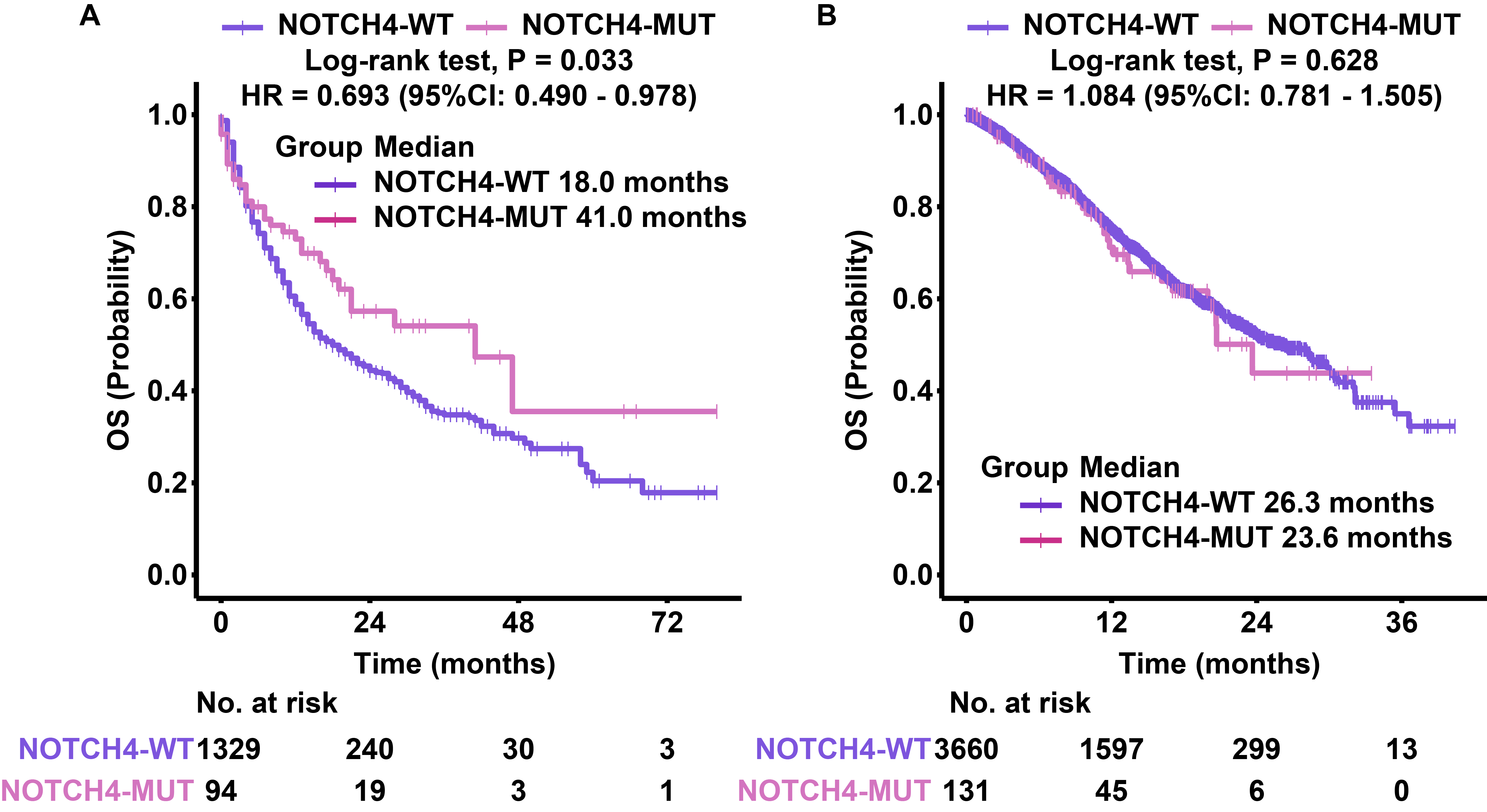

Supplement: Supplementary file 6 — Additional file 6. Figure S4. Association of NOTCH4 status with prognosis in the validation cohort and non-ICI-treated cohort. (A) Predictive value of NOTCH4 mutation for overall survival (OS) in the validation cohort. (B) Predictive value of NOTCH4 mutation for OS in the non-ICI-treated cohort. [file 12916_2021_2031_MOESM6_ESM.tif]

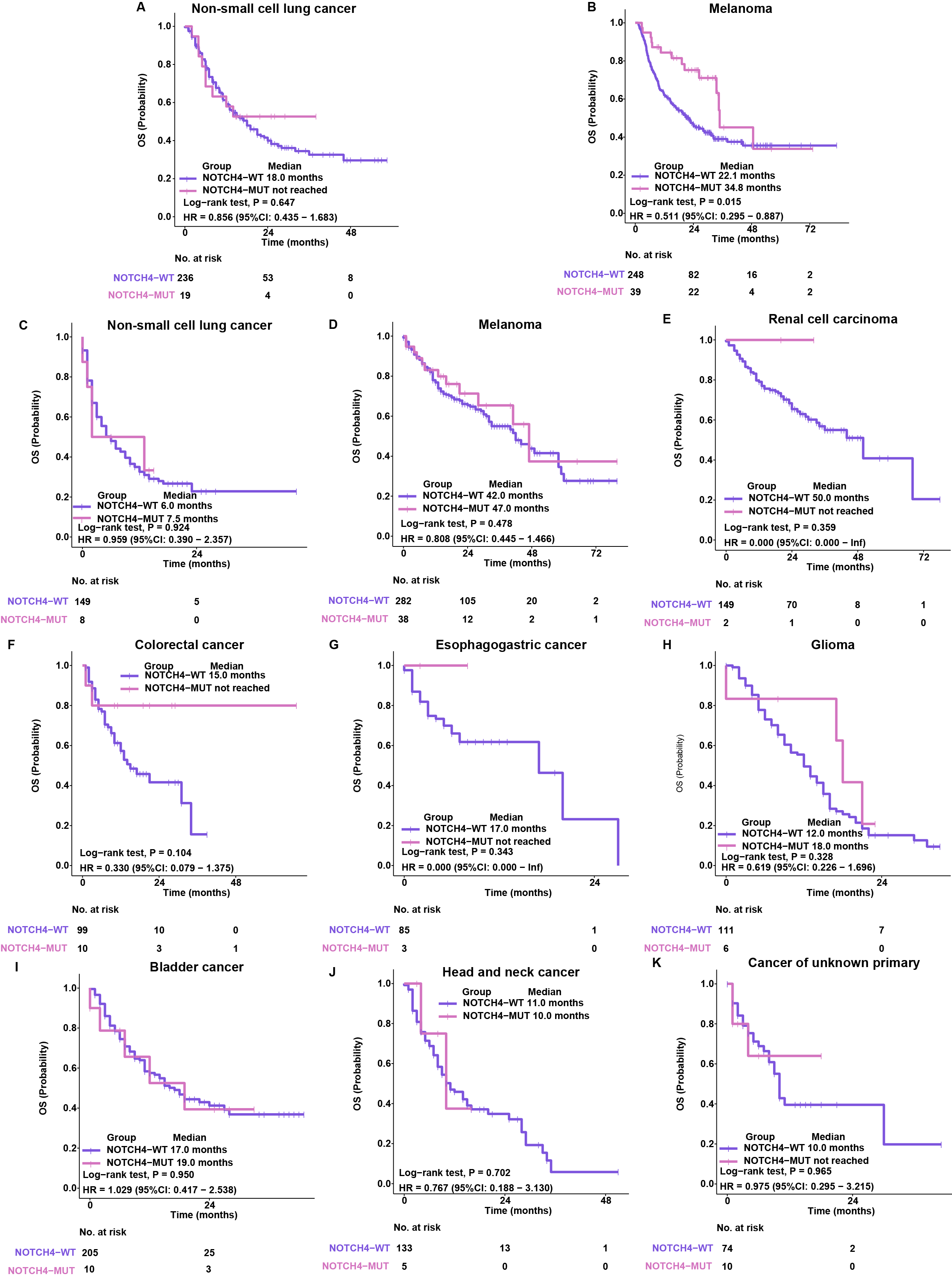

Supplement: Supplementary file 7 — Additional file 7. Figure S5. Subgroup analyses of NOTCH4 status for each cancer type in the discovery cohort and in the validation cohort. (A) Predictive value of NOTCH4 mutation for overall survival (OS) in patients with non-small cell lung cancer in the discovery cohort. (B) Predictive value of NOTCH4 mutation for OS in patients with melanoma in the discovery cohort. (C) Predictive value of NOTCH4 mutation for OS in patients with non-small cell lung cancer in the validation cohort. (D) Predictive value of NOTCH4 mutation for OS in patients with melanoma in the validation cohort. (E) Predictive value of NOTCH4 mutation for OS in patients with renal cell carcinoma in the validation cohort. (F) Predictive value of NOTCH4 mutation for OS in patients with colorectal cancer in the validation cohort. (G) Predictive value of NOTCH4 mutation for OS in patients with esophagogastric cancer in the validation cohort. (H) Predictive value of NOTCH4 mutation for OS in patients with glioma in the validation cohort. (I) Predictive value of NOTCH4 mutation for OS in patients with bladder cancer in the validation cohort. (J) Predictive value of NOTCH4 mutation for OS in patients with head and neck cancer in the validation cohort. (K) Predictive value of NOTCH4 mutation for OS in patients with cancer of unknown origin in the validation cohort. [file 12916_2021_2031_MOESM7_ESM.tif]
