# Supplementary material for: Identification of NOTCH4 mutation as a response biomarker for immune checkpoint inhibitor therapy
Source: BMC Med. 2021 Jul 21;19:154. doi: 10.1186/s12916-021-02031-3 (PMC8293505; doi:10.1186/s12916-021-02031-3)
Supplement: Supplementary file 4 — Additional file 4. Table S2. The activated and repressed genes in the NOTCH pathway. [file 12916_2021_2031_MOESM4_ESM.docx]

**Table S2. The activated and repressed genes in the NOTCH pathway.**

| Pathway | Symbol |
| --- | --- |
| NOTCH-activated gene | CREBBP |
| NOTCH-activated gene | EP300 |
| NOTCH-activated gene | HES1 |
| NOTCH-activated gene | HES2 |
| NOTCH-activated gene | HES3 |
| NOTCH-activated gene | HES4 |
| NOTCH-activated gene | HES5 |
| NOTCH-activated gene | HEY1 |
| NOTCH-activated gene | HEY2 |
| NOTCH-activated gene | HEYL |
| NOTCH-activated gene | KAT2B |
| NOTCH-activated gene | NOTCH1 |
| NOTCH-activated gene | NOTCH2 |
| NOTCH-activated gene | NOTCH3 |
| NOTCH-activated gene | NOTCH4 |
| NOTCH-activated gene | PSEN2 |
| NOTCH-activated gene | LFNG |
| NOTCH-activated gene | NCSTN |
| NOTCH-activated gene | JAG1 |
| NOTCH-activated gene | APH1A |
| NOTCH-activated gene | FHL1 |
| NOTCH-activated gene | THBS2 |
| NOTCH-activated gene | MFAP2 |
| NOTCH-activated gene | RFNG |
| NOTCH-activated gene | MFAP5 |
| NOTCH-activated gene | JAG2 |
| NOTCH-activated gene | MAML3 |
| NOTCH-activated gene | MFNG |
| NOTCH-activated gene | CNTN1 |
| NOTCH-activated gene | MAML1 |
| NOTCH-activated gene | MAML2 |
| NOTCH-activated gene | PSEN1 |
| NOTCH-activated gene | PSENEN |
| NOTCH-activated gene | RBPJ |
| NOTCH-activated gene | RBPJL |
| NOTCH-activated gene | SNW1 |
| NOTCH-activated gene | ADAM10 |
| NOTCH-activated gene | APH1B |
| NOTCH-activated gene | ADAM17 |
| NOTCH-activated gene | DLK1 |
| NOTCH-activated gene | DLL1 |
| NOTCH-activated gene | DLL3 |
| NOTCH-activated gene | DLL4 |
| NOTCH-activated gene | DNER |
| NOTCH-activated gene | DTX1 |
| NOTCH-activated gene | DTX2 |
| NOTCH-activated gene | DTX3 |
| NOTCH-activated gene | DTX3 L |
| NOTCH-activated gene | DTX4 |
| NOTCH-activated gene | EGFL7 |
| NOTCH-repressed gene | ARRDC1 |
| NOTCH-repressed gene | CNTN6 |
| NOTCH-repressed gene | KDM5A |
| NOTCH-repressed gene | NOV |
| NOTCH-repressed gene | NRARP |
| NOTCH-repressed gene | ITCH |
| NOTCH-repressed gene | SPEN |
| NOTCH-repressed gene | FBXW7 |
| NOTCH-repressed gene | HDAC2 |
| NOTCH-repressed gene | CUL1 |
| NOTCH-repressed gene | NCOR1 |
| NOTCH-repressed gene | NCOR2 |
| NOTCH-repressed gene | HDAC1 |
| NOTCH-repressed gene | NUMB |
| NOTCH-repressed gene | CIR1 |
| NOTCH-repressed gene | NUMBL |
| NOTCH-repressed gene | RBX1 |
| NOTCH-repressed gene | SAP30 |
| NOTCH-repressed gene | SKP1 |
| NOTCH-repressed gene | CTBP1 |
| NOTCH-repressed gene | CTBP2 |
